# Rice DST transcription factor negatively regulates heat tolerance through ROS-mediated stomatal movement and heat-responsive gene expression

**DOI:** 10.3389/fpls.2023.1068296

**Published:** 2023-01-31

**Authors:** Yanfei Ding, Mei Zhou, Ke Wang, Aili Qu, Shanshan Hu, Qiong Jiang, Keke Yi, Feijuan Wang, Chong Cai, Cheng Zhu, Zhixiang Chen

**Affiliations:** ^1^ Key Laboratory of Specialty Agri-product Quality and Hazard Controlling Technology of Zhejiang Province, College of Life Sciences, China Jiliang University, Hangzhou, China; ^2^ School of Biological and Chemical Engineering, Ningbo Institute of Technology, Ningbo, China; ^3^ College of Life Sciences, Zhejiang University, Hangzhou, China; ^4^ Key Laboratory of Plant Nutrition and Fertilizer, Ministry of Agriculture, Institute of Agricultural Resources and Regional Planning, Chinese Academy of Agricultural Science, Beijing, China; ^5^ Department of Botany and Plant Pathology, Purdue University, West Lafayette, IN, United States

**Keywords:** plant heat tolerance, DST transcription factor, reactive oxygen species, stomatal aperture, heat shock genes, reproductive traits under heat stress

## Abstract

Plants are frequently subjected to a broad spectrum of abiotic stresses including drought, salinity and extreme temperatures and have evolved both common and stress-specific responses to promote fitness and survival. Understanding the components and mechanisms that underlie both common and stress-specific responses can enable development of crop plants tolerant to different stresses. Here, we report a rice *heat stress-tolerant 1* (*hst1*) mutant with increased heat tolerance. *HST1* encodes the DST transcription factor, which also regulates drought and salinity tolerance. Increased heat tolerance of *hst1* was associated with suppressed expression of reactive oxygen species (ROS)-scavenging peroxidases and increased ROS levels, which reduced water loss by decreasing stomatal aperture under heat stress. In addition, increased ROS levels enhanced expression of genes encoding heat shock protein (HSPs) including HSP80, HSP74, HSP58 and small HSPs. HSPs promote stabilization of proteins and protein refolding under heat stress and accordingly mutation of *HST1* also improved reproductive traits including pollen viability and seed setting under high temperature. These results broaden the negative roles of DST in abiotic stress tolerance and provide important new insights into DST-regulated tolerance to diverse abiotic stresses through both shared and stress-specific mechanisms.

## Introduction

As sessile organisms, plants are constantly exposed to a variety of abiotic stresses such as drought, salinity and heat and have evolved complex and diverse responses to promote survival, growth and development under stress conditions (Zhu, 2016). Over the past several decades, important progress has been made in establishing the core mechanisms of responses and adaptation to major abiotic stresses in plants (Zhang et al., 2022). The best characterized mechanism of drought stress responses is the reduction of water loss through stomatal closure in response to water deficiency. The phytohormone abscisic acid (ABA), which is rapidly induced in leaves under drought stress, plays a central role in drought-induced stomatal closure by activating plasma membrane calcium ion (Ca^2+^) channels, resulting in elevation of cytosolic Ca^2+^ levels (Pei et al., 2000; Jannat et al., 2011; Brandt et al., 2015). This Ca^2+^ elevation causes the efflux of K^+^ and Cl^-^ and the removal of organic solutes from guard cells, resulting in reduced cellular turgor and rapid stomatal closing (Macrobbie, 2000; Schroeder et al., 2001; Song and Matsuoka, 2009). Under high salt levels (primarily Na^+^), plants use the so-called Salt-Overly-Sensitive (SOS) pathway to promote salt tolerance. In this pathway, the Ca^2+^-binding protein SOS3 senses salt stress-elicited Ca^2+^ signal and activates the SOS2 protein kinase, which in turn can phosphorylate and activate the SOS1 Na^+^/H^+^ antiporter at the plasma membrane to extrude Na^+^ from root cells into the soil and into the xylem for long transport to leaves (Zhu, 2002; Quan et al., 2007; Zhu, 2016). Under high temperature, on the other hand, denatured and misfolded proteins accumulate and can lead to proteotoxicity (Izumi, 2019). An important part of heat stress responses universally found in different types of living organisms is the rapid expression of genes encoding heat shock proteins (HSPs) (Craig et al., 1993; Georgopoulos and Welch, 1993; Jakob et al., 1993; Arrigo, 2005; Latijnhouwers et al., 2010; Xu et al., 2012; Reddy et al., 2014). In plants, well-characterized HSPs include Hsp101, Hsp70 and small HSPs. These HSPs act as molecular chaperones that promote folding and refolding of nonnative proteins (Baniwal et al., 2007; Kotak et al., 2007; Von Koskull-Doring et al., 2007; Schramm et al., 2008). HSPs can also monitor misfolded/damaged proteins and target their degradation by the ubiquitin proteasome system, autophagy and other pathways (Arias and Cuervo, 2011; Amm et al., 2014).

Abiotic stresses can occur singularly or together, particularly under natural environmental conditions. Heat stress occurs often with drought and, therefore, has a special relationship with water status in plants (Fahad et al., 2017). Heat stress can also directly or indirectly perturb leaf water status and root hydraulic conductivity as observed in *Lotus creticus* and tomato guard cells (Morales et al., 2003; Banon et al., 2004). Different types of adverse environmental conditions may also cause the same or similar physiological stresses in plant cells and can induce common signaling pathways and responses. For example, both drought and salinity can induce hyperosmotic stress in plant cells, which induces Ca^2+^ signaling, ABA accumulation, stomatal closure and stress-responsive gene expression (Zhang et al., 2022). Various abiotic stresses are also associated with the production of reactive oxygen species (ROS), including superoxide and H_2_O_2_ (Mittler et al., 2022). ROS are produced from a variety of sources in plant cells under stress and are highly toxic at high levels. As a result, induction of ROS-scavenging activities is an important and common plant response to different stresses. However, ROS also play important role in stress signaling (Mittler et al., 2022). In guard cells, ROS including H_2_O_2_ act as second messengers for ABA in the regulation of stomatal movement by activating plasma membrane Ca^2+^ channels, resulting in elevation of cytosolic Ca^2+^ levels (Pei et al., 2000; Jannat et al., 2011; Brandt et al., 2015). Under heat stress, ROS, especially H_2_O_2_, are continuously produced in plant cells and the redox balance of cells is disturbed leading to oxidative damage (Apel and Hirt, 2004). On the other hand, heat-induced ROS can also trigger the mobilization and activation of heat shock transcription factor A to promote expression of heat-responsive genes including those encoding HSPs (Giesguth et al., 2015). ROS also regulate stomatal movement to control rates of transpiration, thereby reducing heat-induced water loss (Singh et al., 2017; Qi et al., 2018). Dissection of the complex mechanisms responsible for shared and coordinated stress responses could provide new insights into the comprehensive networks of plant stress responses. This knowledge is also necessary to engineer plants with tolerance to different abiotic stresses by targeting common mechanisms and core pathways of plant stress responses.

Rice is the most important grain that provides more than one-fifth of the calories consumed worldwide by humans. Even though rice thrives in hot climates, extreme heat can irreversibly damage the crop, particularly during germination and fertilization, causing loss in yield and grain quality. To identify genes required for rice heat tolerance, we have performed large-scale screens of a rice T-DNA insertion population for mutants with altered heat tolerance and isolated the *heat stress tolerance 1* or *hst1* mutant. Through positional cloning, we have isolated the *HST1* gene and found it encoding a Cys-2/His-2-type (C2H2) zinc finger transcription factor. Interestingly, rice *HST1* was isolated initially as *Drought and Salt Tolerance* (*DST*) for its negative role in drought and salt tolerance and more recently as *Regulator of Gn1a 1* (*REG1*) for its negative role in the regulation of grain number (Huang et al., 2009; Li et al., 2013b; Cui et al., 2015). Unlike the previously reported *dst1* and *reg1* mutants that still produce mutant DST proteins capable of binding DNA, *hst1* is a complete loss-of-function mutant. Based on the increased heat tolerance of the *hst1* mutant, we have hypothesized that HST1 is an important regulator of rice responses to multiple types of abiotic stresses through both common and distinct molecular mechanisms. To test this hypothesis, we have comprehensively characterized the *hst1* mutant for its role in heat tolerance at both seedling and reproductive stages. We have also analyzed the contribution of drought-related mechanisms such as ROS-mediated stomatal and water status (Huang et al., 2009; Cui et al., 2015) as well as heat-induced mechanisms to the enhanced heat tolerance of the *hst1* mutant. These studies have provided important new insights into the broad role of the transcription factor in rice stress tolerance.

## Materials and methods

### Rice growth and treatment

Rice cultivar Zhonghua 11 is a heat sensitive japonica variety and was used as WT throughout the study. The *hst1* mutant was isolated from the T_7_ generation of ZH 11 T-DNA insertion mutant lines. Rice seeds were sterilized with 10% (v/v) sodium hypochlorite for 15 min before rinsing five times with sterilized water, then soaked in the water at 37°C for 2 d in the dark. After germination in Petri dishes with wet filter paper at 37°C, the most uniformly germinated seeds were transferred to a 96-well plate, from which the bottom was removed. The plate was floated on water for 5 days in a growth chamber with a 13-h light (28°C)/11-h dark (23°C) photoperiod and 65% relative humidity. After 4 days, the seedlings were cultured with Yoshida’s culture solution (Yoshida et al., 1976). For heat-tolerant screening, rice seedlings were subjected to treatment of heat stress at 42°C with a 13h light/11h dark photoperiod.

### Analysis of heat and salt tolerance

For heat treatment at the vegetative stage, WT and *hst1* mutant seedlings that were grown hydroponically in 4.5 liters of Yoshida’s culture solution were transferred to a chamber and treated for 3 days at 42°C with a 13h light/11h dark photoperiod. The plants were then transferred to the normal temperature for recovery. The daily loss of the volume of Yoshida’s culture solution was very small (<5%) and was replenished with water daily during the heat treatment and recovery period. The heat tolerance phenotypes of the plants were examined, and plants were photographed after different days of recovery at normal temperature. For analysis of the effect of heat stress on reproductive performance, rice plants were moved to a growth chamber one day prior to heading and subjected to heat treatment for 7 days with a 13h light/11h dark photoperiod. The heat treatment (40°C) lasted for 6 hours daily during the light period from 9:00AM to 15:00PM. The plants were grown at 30°C for the remaining 7 hours (3 and 4 hours before and after heat treatment, respectively). Control plants were grown at 28°C during the 13-hour light period. All plants were grown at 23°C during the 11-hour dark period. Plants were grown under normal growth conditions after treatment and their reproductive traits were evaluated after they reached full maturity.

For salt treatment, WT and *hst1* mutant seedlings grown in pots under normal conditions were transferred to plastic containers containing Yoshida nutrient solution with 0.6% NaCl for 12 days. After the treatment, the plants were transferred to the normal nutrient solution for recovery.

### Map-based cloning approach

The *hst1* mutant was crossed with IR29, a heat-sensitive indica rice variety. All F1 progeny showed a heat-sensitive phenotype. In the F2 population, heat-sensitive plants and heat tolerant plants segregated in a ratio close to 3 to one. Preliminary mapping with about 300 F2 plants located the *hst1* gene on chromosome 3 using PCR-based molecular markers (**Supplemental Table 1**). Further mapping placed the mutant gene in a region on chromosome 3. The *hst1* gene was putatively identified by sequencing the genes in the region and confirmed by complementation with the WT *HST1* gene.

### *HST1* gene cloning and rice transformation

For complementation of the *hst1* mutant, a 3970-bp DNA fragment of the full-length *HST1* genomic sequence was amplified from genomic DNA and cloned into pCAMBIA1301 with *Kpn* I and *Sal* I enzymes. Transgenic lines were achieved by co-cultivation of *hst1* rice calli with *Agrobacterium tumefaciens* strain EHA105 containing the *HST1* construct (Hiei et al., 1994). Positive T0 transgenic plants were screened using PCR analysis of *hpt* with genomic DNA from their leaves.

### Production of recombinant DST proteins and EMSA

Full-length coding sequences of WT DST and mutant DST^hst1^ proteins were amplified by PCR using gene-specific primers and cloned into the expression vector pET-32a (Novagen) and transformed into *E. coli* strain BL21(DE3). Expression and purification of recombinant proteins were performed as previously described (Lai et al., 2011). EMSA was performed with purified recombinant proteins and ^32^P-labeled double-stranded synthetic oligonucleotides as described previously (Lai et al., 2011).

### Water loss and relative water content assay

Water loss from the detached leaves of WT plants and *hst1* mutants was measured according to the method described by (Tian et al., 2004) with minor modifications. Detached leaves were placed at 24°C with 40% relative humidity and their fresh weights were determined at various time points. Water loss was expressed as the percentage of initial fresh weight at each time point. The measurement of relative water content was performed as described (Ascenzi and Gantt, 1999) with slight modifications. Briefly, eight fully expanded leaves were detached from plants after various periods of heat treatment and immediately determined for the fresh weight. The leaves were then submerged in ddH_2_O overnight. Water on the surface of the leaves was removed by quick blotting using filter papers and the turgid weight was determined immediately. The leaves were then dried at 105°C for 4 hours before their dry weight was determined.

### Stomatal conductance and density measurement

For assays of stomatal conductance, 10-day-old plants were grown in a growth chamber. The stomatal conductance was measured using a portable photosynthesis system (LI-6400 LI-COR, Lincoln, USA). For stomatal density measurement, middle sections of fully expanded leaves were sampled from the same position of rice plants of WT and *hst1* mutant. A leaf surface imprint method was used. Nail polish was painted on the sample. After the nail polish was dry, the smear layer was obtained and spread onto the glycerin and observed under a light microscope as previously described (Berger and Altmann, 2000).

### Peroxidase activity, H_2_O_2_ and malondialdehyde level detection

The peroxidase activity was measured using a peroxidase assay kit from Nanjing Jiancheng Bioengineering Institute (Nanjing, China) as described previously (Li et al., 2013a). H_2_O_2_ content was measured using an Amplex^®^ Red Hydrogen Peroxide/Peroxidase Assay Kit (Invitrogen) following the vendor’s instructions. For sample preparation, 0.5g leaf segments of 10-day-old seedlings were ground in liquid nitrogen and thoroughly mixed with 10 volumes of 50 mM Na_3_PO_4_ (pH 7.4). After centrifugation at 12000 rpm for 20 minutes at 4 °C, the supernatants were used for the H_2_O_2_ assay. The levels of MDA were determined based on the spectrophotometer measurement of the product from the reaction of MDA and thiobarbituric acid as previously described (Li et al., 2013a).

### RNA isolation and qRT-PCR analysis

Total RNA was isolated using Trizol reagent (Invitrogen) from WT and *hst1* mutant seedlings and flowers. Total RNA was treated with 5 U of RNase-free DNase I (TaKaRa) to remove any DNA contamination. DNase I-treated RNA was reverse transcribed using an oligo(dT) primer and a PrimeScript RT reagent kit (TaKara, Japan) to generate cDNA. Real-time PCR was carried out using SYBR Premix Ex Taq (TaKara, Japan) for detection of PCR products (parameters: 95°C for 1 min, followed by 40 cycles of 95°C for 15 s, 60°C for 15 s, and 72°C for 15 s). All reactions were performed in triplicate. Quantification of gene expression was done using the comparative Ct method. The gene encoding *ACTIN* (LOC_Os03g50885) was chosen as a reference gene. The primers used for qRT-PCR were shown in **Supplemental Table 2**.

### Western blotting

Total proteins from rice leaf tissues were extracted with P-PER^®^ Plant Protein Extraction Kit (Pierce, Cat # 89803). Proteins were fractionated by SDS-PAGE and the HSP70 protein levels were detected by western blotting for detection using an anti-HSP70 antibody (Abcam, Cat # ab5439). The Rubisco large subunit, which was detected with a Rubisco-specific antibody (Agrisera, Cat # AS07 259), was used an loading control. The antigen-antibody complexes were detected by enhanced chemiluminescence using luminal as substrate. The western blot films were scanned, and the intensities of protein bands were quantified using the Image J software. The quantification reflected the relative amounts as a ratio of each HSP protein band to the lane’s Rubisco loading control.

### Pollen viability assays

Pollen viability was tested with the peroxidase reaction method as described previously (Wang et al., 2012). Briefly, pollens were collected onto a glass slide and stained with two reagents (reagent I: 0.5% benzidine, 0.5% *α*-naphthol and 0.25% sodium carbonate at 1:1:1 ratio and reagent II: 0.3% hydrogen peroxide). After incubation at 30°C for 10 minutes, the pollen viability was examined under a microscope with viable pollens stained blue and unviable pollens stained colorless or yellowish.

## Results

### Isolation of the rice *hst1* mutant

**Figure 1:**
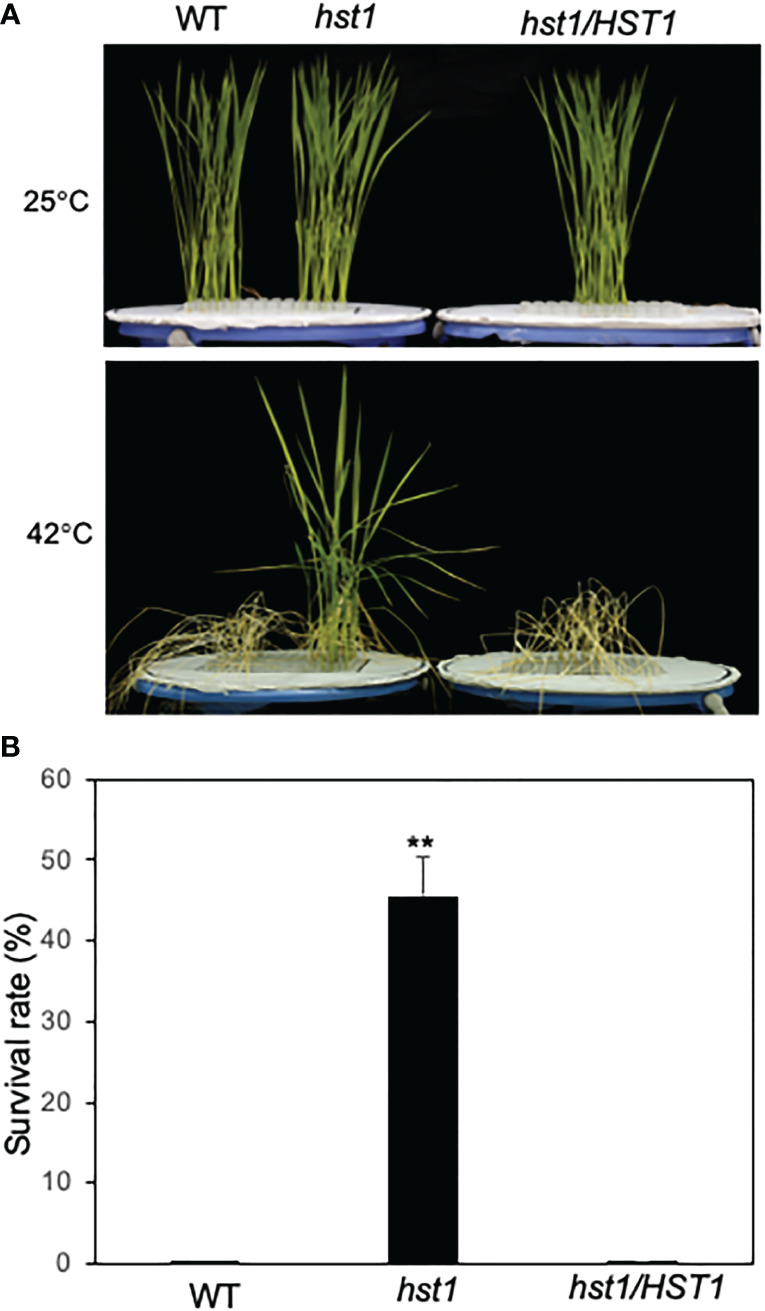
Enhanced heat tolerance of rice WT and *hst1* mutant. **(A)** Survival of rice plants after heat treatment. Ten-day old rice seedlings of WT, *hst1* mutant and *hst1* mutant containing a full length HST1 genome clone (*hst1/HST1*) were subjected to heat treatment for 3 days at 42°C. The pictures were taken after 12 days of recovery after heat treatment. The pictures of plants grown at 25°C are also shown for comparison. **(B)** Plant survival rates of WT, *hst1* mutant and *hst1* mutant containing a full length HST1 genome clone (*hst1/HST1*) after heat treatment. Means and SE were calculated from survival rates determined from three experiments with about 100 seedlings per experiment for each genotype. The statistical differences in the survival rate between WT and *hst1* mutant plants were tested using a Student *t* test (**P ≤ 0.01).

To gain important insights into the genes and their network in controlling heat stress responses in rice, we performed a large-scale screen of the rice ZH11 T-DNA insertion lines for mutants with altered levels of wilting immediately after heat stress, or survival after recovery at normal temperature. We isolated several mutants that showed increased heat tolerance and retesting of these putative heat-stress-tolerant mutants confirmed one of them to be highly heat-tolerant in the subsequent generations and was named *heat-stress tolerant 1* (*hst1*). After heat treatment at 42°C for 3 days (13 h light/11h dark photoperiod), the *hst1* mutant seedlings showed less severe wilting than wild-type (WT) plants (data not shown). After 12 days of recovery at 25°C following the heat treatment, approximately 44% of the *hst1* mutant seedlings survived but almost all of WT seedlings died (**Figure 1**). Based on the wilting symptoms and survival rates after heat treatment, we concluded that the *hst1* mutant had substantially improved heat tolerance.

### Cloning and characterization of *HST1*

Since the *hst1* mutant was isolated from a rice T-DNA insertion population, we first determined whether the mutation was caused by a T-DNA insertion. The T-DNA insertion in the binary vector contains a *hygromycin phosphotransferase* (*hpt*) gene as a selection marker that confers resistance to the antibiotic hygromycin. Therefore, we first determined resistance of *hst1* to hygromycin and, surprisingly, discovered that the mutant was as sensitive to the antibiotic as WT plants. To ensure that the hygromycin sensitivity was not due to silencing of the selection marker, we also performed PCR using *hpt*-specific primers but failed to amplify the antibiotic resistance gene from the mutant. Therefore, the *hst1* mutant plant is unlikely to be a T-DNA insertion line. To further determine the genetic nature of the *hst1* mutant, we crossed the mutant with WT plants and found *hst1* to be a recessive mutation based on phenotyping of their F1 and F2 progeny. These results collectively suggested that the *hst1* mutant resulted from a loss-of-function mutation rather than by a T-DNA insertion.

**Figure 2:**
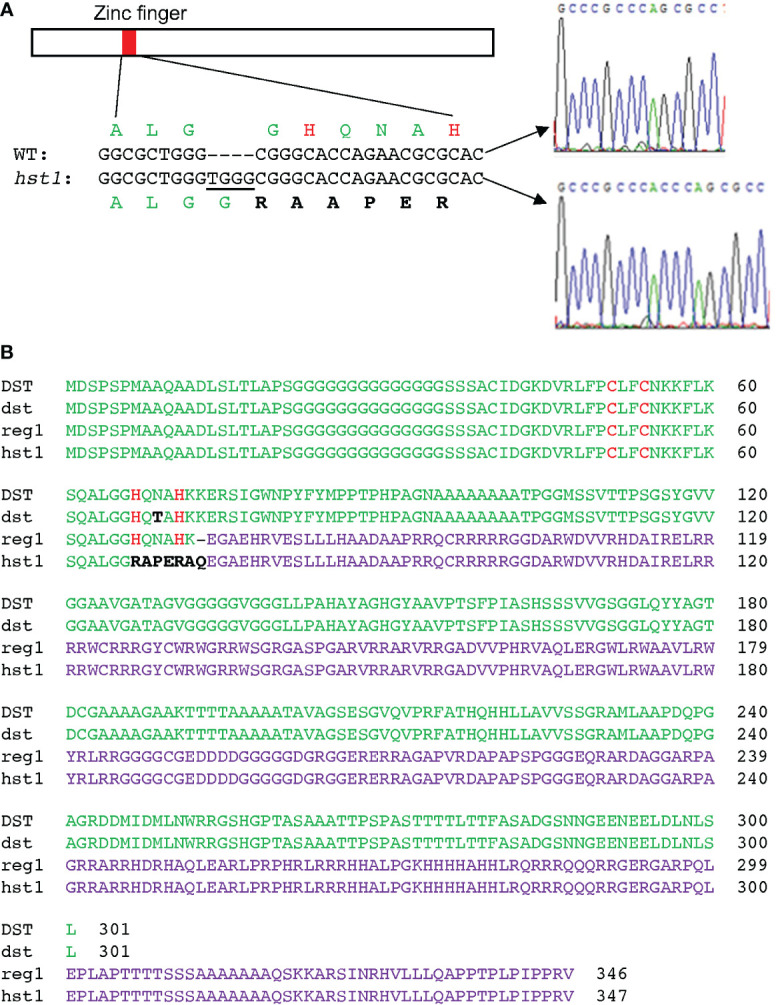
Positional cloning and gene product of the *hst1* mutant gene. **(A)** Identification of the *hst1* gene mutation. The *hst1* gene contains a four-nucleotide (TGGG) insertion at the nucleotide position 194, which leads to a frameshift of the translated protein at its CCHH zinc finger motif. **(B)** Comparison of WT DST protein with the three isolated mutant proteins: DST^dst^ (dst), DST^reg1^(reg1) and DST^hst1^(hst1). The dst protein contains a single amino acid substitution (N69T) between the two conserved H residues at the zinc finger motif. Both *reg1* and hst1 proteins result from frameshift mutations that alter a majority of the DST amino acid sequence. The reg1 protein still contains the intact zinc finger motif while the hst1 protein lost the conserved histidine H residues of the zinc finger motif due to the frameshift mutations. The conserved zinc-binding cysteine (C) and H residues of the zinc finger motif are in red. The unique amino acid residues in dst and hst1 from their respective mutations are in black. The altered C-terminal amino acid sequences from reading frame shifting in reg1 and hst1 proteins are in purple.

We then took a map-based cloning approach to isolate the *HST1* gene. We first crossed the *hst1* mutant (as female) with a heat sensitive Indica rice variety IR29 (as male parent). All F1 progeny showed a WT heat-sensitive phenotype based on the survival and recovered growth after heat treatment. In the F2 population, the ratio of the heat-sensitive plants to the heat-tolerant plants was close to 3 (220 heat-sensitive *vs*. 80 heat-tolerant; x^2 =^ 0.444<x^2^_0.5_^=^ 0.455; P>0.5), indicating that the *hst1* mutant was caused by a single recessive nuclear gene mutation. The *HST1* locus was fine-mapped to a region of approximately 80 kb on chromosome 3 (**Supplemental Figure 1**). The genes within the region were sequenced, and it was discovered that one of them (LOC_Os03g57240) contains a four-nucleotide insertion (TGGG) between the 194^th^ and 195^th^ nucleotides of its coding sequence in the *hst1* mutant (**Figure 2A**). The insertion would lead to a frameshift in translation after the first 64 amino acid residues (**Figure 2**). To confirm that LOC_Os03g57240 is *HST1*, we performed a complementation test. A 3966-nucleotide WT LOC_Os03g57240 genomic fragment containing both its promoter and coding region was amplified and cloned into the binary vector pCAMBIA1300. The recombinant vector pCAMBIA1300-*HST1* was transformed into the callus of the *hst1* mutant by an *Agrobacterium*-mediated method. Five positive transgenic *hst1/Hst1* lines were obtained and were as heat-sensitive as WT based on increased wilting and reduced survival rate when compared to the *hst1* mutant after heat treatment (**Figure 1**). Thus, the TGGG insertion in LOC_Os03g57240 is responsible for the increased heat-tolerant phenotype of the *hst1* mutant. These results indicate that HST1 has a negative role in heat tolerance in rice.

**Figure 3:**
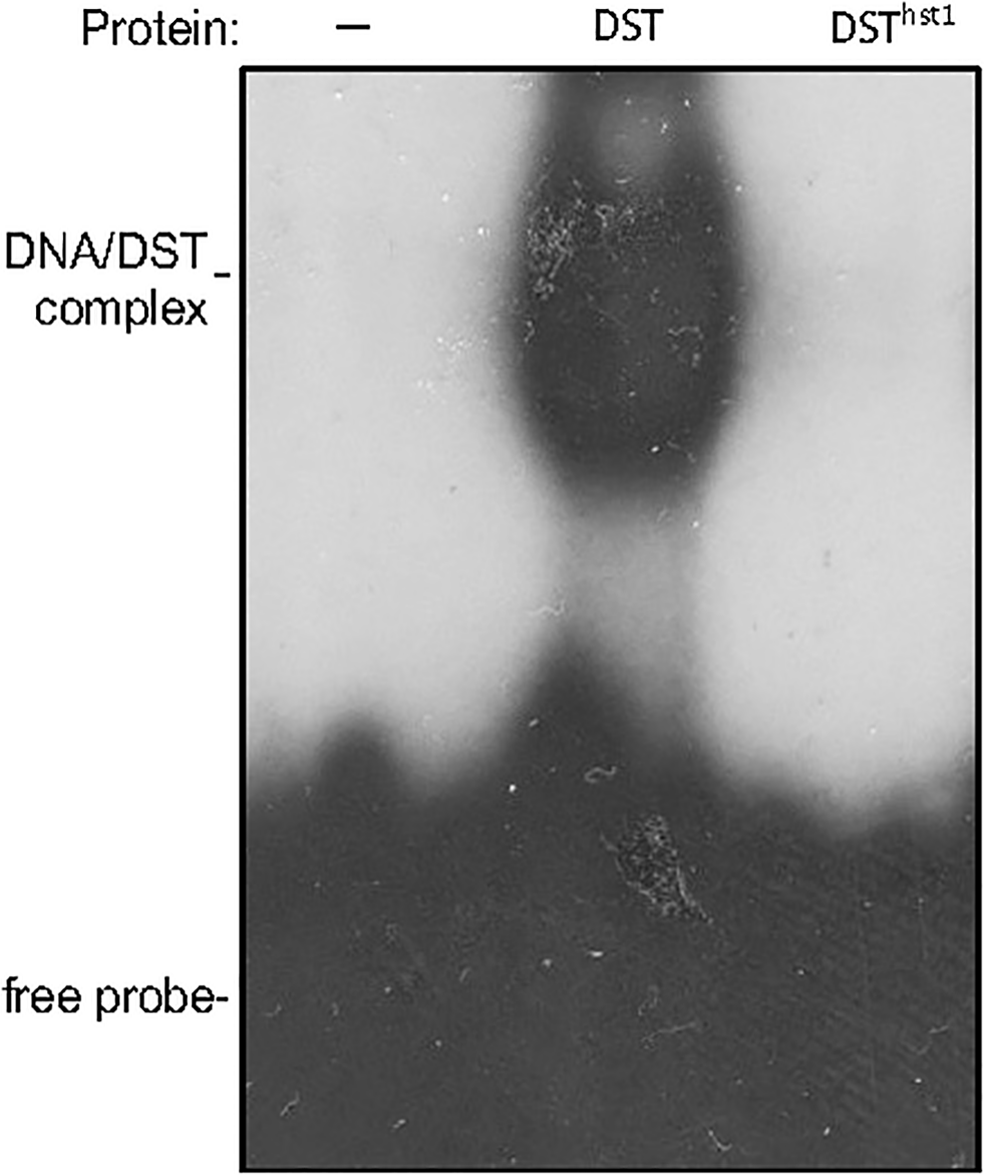
Assays of DNA-binding activity of DST and DST^hst1^ proteins. Binding reactions (20 μl) contained no (-) or 0.5 μg indicated recombinant proteins, 2 ng labeled oligo DNA (GGCTATACTAACCGTGCtgctagccattagGCCCAAGTTAGT) and 5 μg polydeoxyinosinic-deoxycytidylic acid.

Interestingly, LOC_Os03g57240 was previously named *Drought and Salt Tolerance* (*DST*), which negatively regulates drought and salt tolerance in rice plants based on the analysis of a *dst* mutant (Huang et al., 2009; Cui et al., 2015). The *DST* gene encodes a 301-residue transcription factor containing a nuclear localization signal (NLS) and a C2H2-type zinc finger motif at the N-terminus. The C2H2-type zinc finger of DST is responsible for its sequence-specific DNA binding activity (Huang et al., 2009; Li et al., 2013b; Cui et al., 2015). The *dst* mutant is recessive with increased drought and salt tolerance due to a missense mutation that converted the codon for asparagine at residue 69 to a codon for threonine in the conserved C2H2 zinc finger motif (**Figure 2B**) (Huang et al., 2009). A second mutant, *reg1*, is semidominant with increased panicle branches and consequently improved grain number (Li et al., 2013b). The *reg1* mutant contains an A insertion between the 214^th^ and 215^th^ nucleotides of the *DST* cDNA, which leads to a frameshift after the first 72 amino acids (**Figure 2B**) (Li et al., 2013b). As a result, the NLS and C2H2 zinc finger motif at the N-terminus of DST^reg1^ are not altered but the remaining protein sequence is completely changed as a result of the frameshift (**Figure 2B**) (Li et al., 2013b). Both DST^dst^ and DST^reg1^ mutant proteins still contain the conserved C2H2 residues in the zinc finger motif and, as a result, still bind to the TGNTANN(A/T)T sequence, a *cis*-acting element called DST-binding sequence present in DST target genes (Huang et al., 2009; Li et al., 2013b; Cui et al., 2015). By contrast, the two conserved histidine residues in the DNA-binding C2H2 zinc finger motif in our DST^hst1^ mutant protein are eliminated by the frameshift mutation in the *hst1* mutant (**Figure 2**), which would abolish its sequence-specific DNA-binding activity. To test this, we produced recombinant DST and DST^hst1^ proteins and analyzed them for sequence-specific DNA-binding activity using electrophoresis mobility shifting assays (EMSA). As shown in **Figure 3**, only WT DST but not the DST^hst1^ protein was able to bind DNA molecules containing the DST-binding sequence.

### Expression analysis of rice *HST1(DST)*

**Figure 4:**
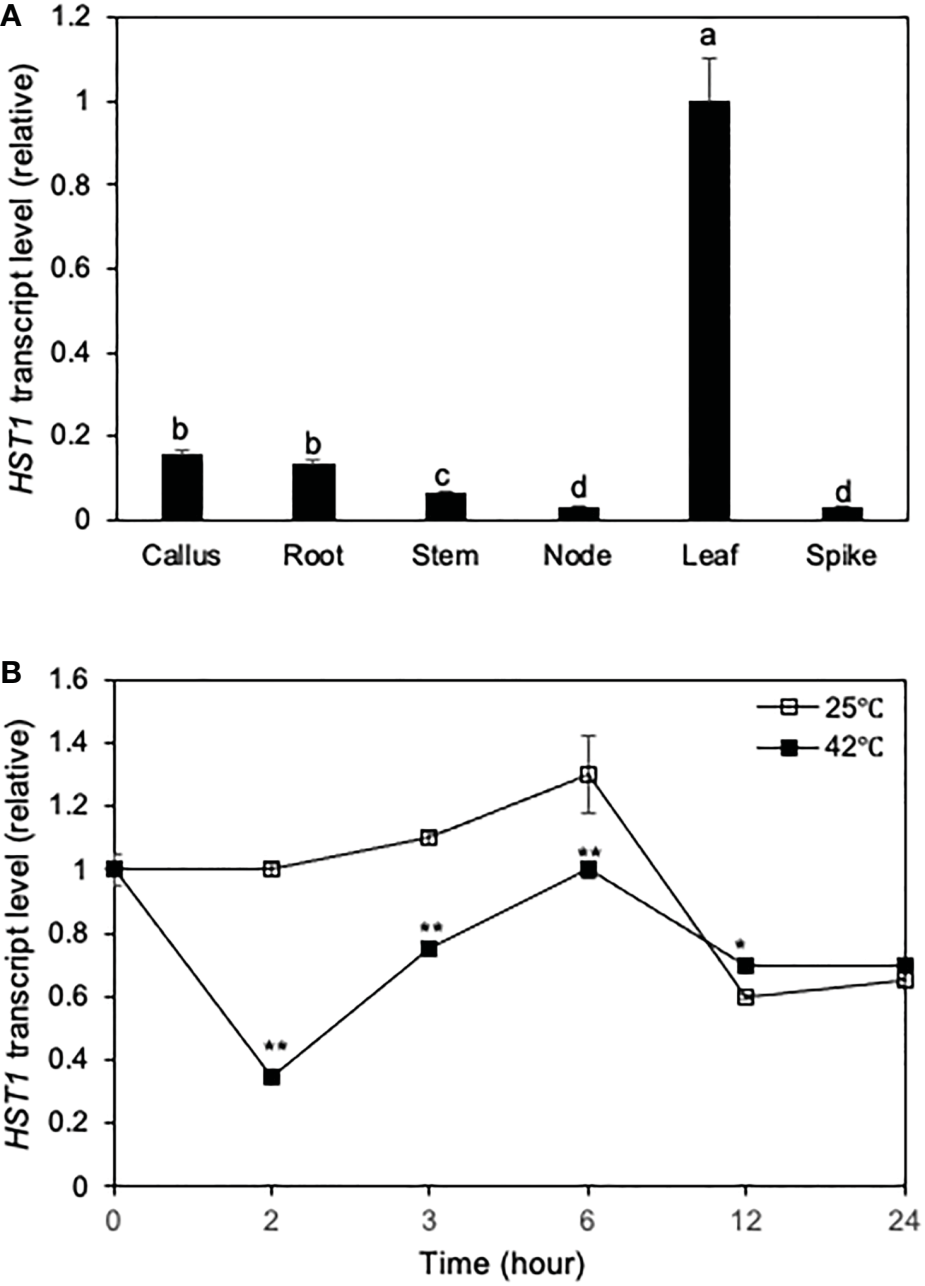
Tissue-specific and heat-regulated expression of *DST*. **(A)** Tissue-specific expression of *DST*. Total RNA was isolated from indicated tissues and transcript levels of *DST* were determined using qRT-PCR. Error bars indicate SE (n = 3). According to Duncan’s multiple range test (P=0.01), means of the values do not differ if they are indicated with the same letter. **(B)** *DST* gene expression in response to heat treatment. Rice seedlings (10-days old) were placed in a 25°C or 42°C growth chamber and total RNA was isolated from leaf samples collected at indicated times. Transcript levels of *DST* were determined using qRT-PCR. Error bars indicate SE (n = 3). The statistical differences in the transcript levels between 25°C and 42°C were tested using a Student *t* test (*P ≤ 0.05; **P ≤ 0.01).

To further analyze the role of the rice *DST* gene, we analyzed its tissue-specific expression using qRT-PCR. As shown in **Figure 4A**, although *DST* transcripts were detected in all tissues, their levels were relatively low in callus, root, stem, node and spike. On the other hand, high levels of *DST* transcripts were detected in leaves (**Figure 4A**). As a negative regulator of plant responses to a range of abiotic stresses, high levels of DST transcripts in rice leaves may be necessary to suppress induction of stress responses under normal conditions and promote other processes such as photosynthesis in the green tissues that are important for plant growth.

We also analyzed the expression pattern of *DST* in rice leaves in response to heat stress. qRT-PCR analysis showed that the levels of *DST* transcripts were rapidly down-regulated after 1 hour of heat treatment, followed by gradual recovery over the subsequent 6 hours (**Figure 4B**). During the first 6 hours of heat treatment, the transcript levels of *DST* in heat-treated leaves were generally lower than those in the leaves with heat treatment (**Figure 4B**). Thus, heat stress reduced expression of *DST* during the first 6 hours of heat treatment. After 6 hours, the *DST* transcript levels decreased not only at 42°C but also at 25°C (**Figure 4B**). This reduction in *DST* transcripts was likely in response to the light-to-dark transition of the rice plants.

### Altered water and stomatal status of *hst1* under heat stress

**Figure 5:**
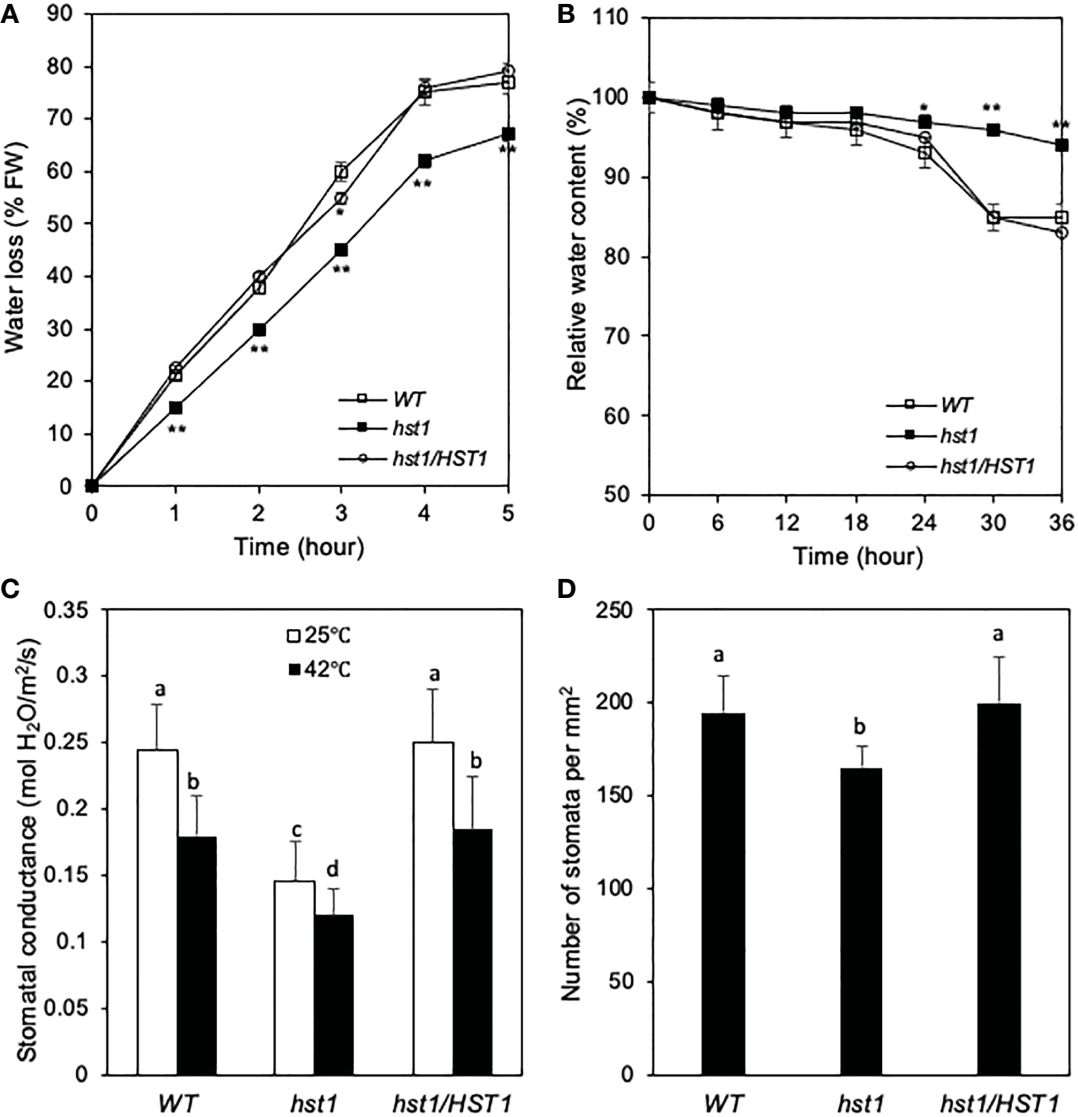
Effects of the *hst1* mutation on leaf stomata and water loss under heat stress. **(A)** Water loss of detached leaves. For each repeat, 10 fully expanded leaves of 10-day-old plants were detached from WT and the *hst1* mutant plants and placed at 24°C with 40% relative humidity in a triplicate experiment (n = 3). **(B)** Relative water content. Relative water content of WT and *hst1* mutant treated with heat (42°C) at 65% relative humidity using the fully expanded leaves of 10-day-old plants (n = 9). **(C)** Stomatal conductance. Seedlings were cultured in a growth chamber for 10 days, and the stomatal conductance was measured using a portable photosynthesis system (LI-6400 LI-COR, Lincoln, USA). The statistical differences in water loss or water content between WT and *hst1* mutant plants including the *hst1* mutant plants containing the full *HST1* genome clone (*hst1*/*HST1*) were tested using a Student *t* test (*P ≤ 0.05; **P ≤ 0.01). **(D)** Stomata number. Stomatal density of middle sections of leaves from WT and *hst1* mutant plants (n = 10) were determined from three random microscopic fields in each repeat. According to Duncan’s multiple range test (P=0.01), means of the values do not differ if they are indicated with the same letter. The experiment was repeated three times with similar results.

To understand the physiological basis of the reduced wilting phenotype of the *hst1* mutant during heat treatment, we analyzed the relative water content in the WT and *hst1* mutant plants. Detached WT and *hst1* mutant leaves were placed at 24°C under 40% relative humidity and water loss was measured after various hours. As shown in **Figure 5A**, WT rice leaves lost 20-38% more water during the first 4 hours. After 4 hours at the high temperature, water loss in the detached WT leaves was substantially reduced likely due to limited amounts of residual water in the leaves. In the *hst1* mutant, water loss also decreased after 4 hours under the heat stress but to a lesser extent than in WT probably due to higher levels of residual water in the mutant leaves (**Figure 5A**). We also subjected whole WT and *hst1* mutant plants to heat stress (42°C) and monitored the relative water content for 36 hours. As shown in **Figure 5B**, significant difference in relative water content between WT and *hst1* plants was first apparent after 24 hours under heat stress and became more pronounced in the next 12 hours. As a result, during the last 8-10 hours of the experiments, the *hst1* mutant plants steadily maintained about 10% more water than the WT plants (**Figure 5B**). Thus, the *hst1* mutant plants were more resistant to water loss than WT plants at the high temperature, which would allow for better survive under heat stress.

Stomata are vital for sensing and adapting to environmental changes including mitigating transpirational water loss under water or other stresses. Therefore, we investigated the stomatal conductance of the *hst1* mutant and WT plants. Even under normal temperature, the stomatal conductance of the *hst1* mutant was only about 60% of that of WT plants (**Figure 5C**). Under heat stress, the stomatal conductance of WT and the *hst1* mutant plants was reduced by about 30% and 20%, respectively, when compared to those under normal temperature (**Figure 5C**). Even with the relatively small reduction for the *hst1* mutant with elevated temperature, the stomatal conductance of the *hst1* mutant was still about 30% lower than that of WT plants under heat stress (**Figure 5C**). Furthermore, we found that the stomatal density in the *hst1* mutant plants was 15% lower than that in WT plants (**Figure 5D**). Thus, the enhanced heat tolerance of *hst* mutants was associated with decreased stomatal density and low stomatal conductance, which would make the mutant retain more water and survive better than WT plants under heat stress.

To determine whether altered water status of the *hst1* mutant was caused by the *DST* gene mutation, we analyzed transgenic *hst1* lines containing the WT *DST^HST1^* genomic fragment (*hst1/HST1*) for relative water content and water loss. Indeed, all transgenic *hst1/HST1* plants were similar to WT with reduced water content and increased water loss after heat treatment when compared to those of the *hst1* mutant (**Figures 5A, B**). Furthermore, the stomatal conductance and stomata number of the *hst1* mutant were restored to those of WT in the complemented lines (**Figures 5C, D**). These results further indicate that the TGGG insertion in the *DST^HST1^* gene is responsible for the increased heat tolerance of the *hst1* mutant, demonstrating that the DST transcription factor is a negative regulator of heat tolerance in rice.

**Figure 6:**
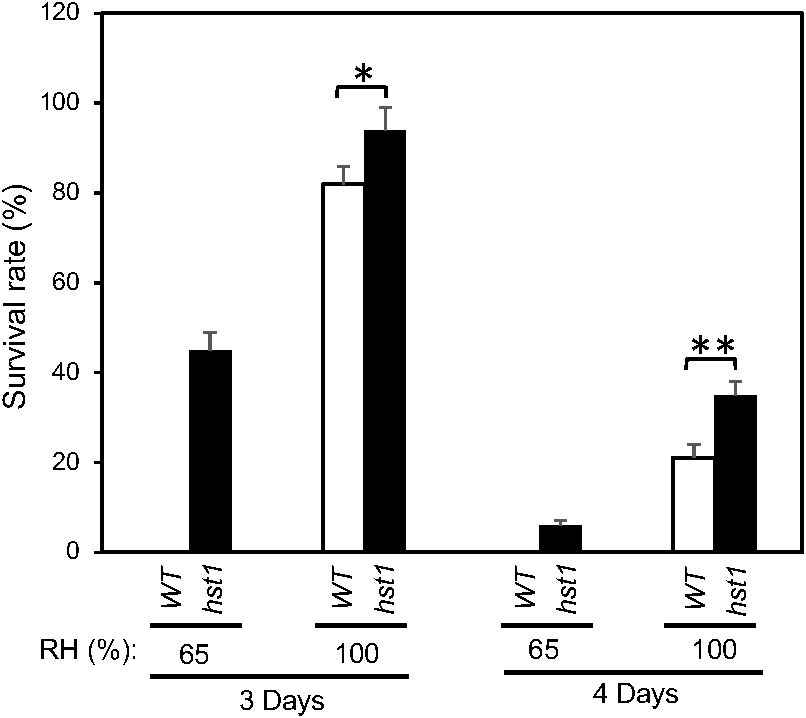
Effects of humidity on the heat tolerance of rice WT and *hst1* mutant. Ten-day old WT and *hst1* mutant plants were subjected to heat treatment at 42°C for 3 and 4 days under 65% and 100% relative air humidity. The survival rates were estimated after 10 days of recovery at the room temperature. Means and SE were calculated from survival rates determined from three experiments with about 50 seedlings per experiment for each genotype. The statistical differences in the maximum leaf width between WT and *hst1* mutant were tested using a Student *t* test (*P ≤ 0.05; **P ≤ 0.01).

To determine whether the enhanced heat tolerance of the *hst1* mutant was entirely or partially due to its reduced stomatal conductance, which reduces water loss under heat stress, we compared survival rates of WT and *hst1* mutant plants after heat treatment under 65% and 100% of relatively air humidity. As shown in **Figure 6**, increased air humidity improved survival rates of both WT and *hst1* mutant plants after both 3 and 4 days of heat treatment. Furthermore, the differences in the survival rates after heat stress between WT and *hst1* mutant plants decreased when the air humidity was increased from 65% to 100% (**Figure 6**). It should be noted that at both 65% and 100% humidity, the rice plants were grown hydroponically in a large volume of growth medium (4.5 liters) that was replenished daily during the heat treatment and recovery period. Therefore, the differences in the survival rates after heat treatment between the two levels of air humidity and between WT and the *hst1* mutant were not caused by reduced water supply or drought. Instead, the substantial effect of relative air humidity on the heat tolerance was mostly through their effect on the water status of shoots above the ground, which is controlled not only by the water supply from the roots but also by water loss from transpiration. Furthermore, even with 100% air humidity, there were still significant differences in the survival rates between WT and *hst1* mutant after 3 or 4 days of heat stress (**Figure 6**). These results indicate that stomatal conductance plays an important role in increased heat tolerance of the *hst1* mutant but other factors also contribute to improved survival of *hst1* under high temperatures.

### Reduced expression of ROS-scavenging *Prx24* target gene and altered H_2_O_2_ homeostasis in the *hst1* mutant

**Figure 7:**
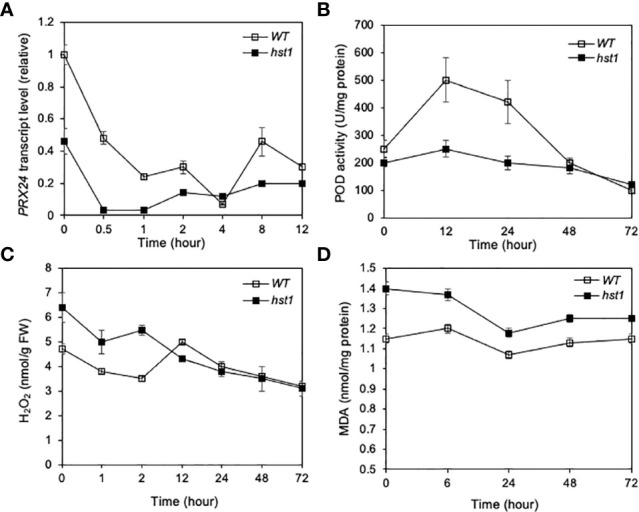
Effect of the *hst1* mutation on expression of ROS-scavenging *Prx24* target gene and levels of ROS and MDA under heat stress. **(A)** Expression of *Prx24*. Rice WT and *hst1* mutant seedlings (10-day old) were placed in a 42°C growth chamber and total RNA was isolated from leaf samples collected at indicated times. Transcript levels of *Prx24* were determined using qRT-PCR. Error bars indicate SE (n = 3). **(B)** Level of peroxidase (POD) activity. Heat treatment of rice seedlings was performed as in (c). One unit (U) POD activity is defined as the amount of enzyme which catalyzes 1 µg substrate in 20 seconds at 25°C. Means and SE were calculated from three experiments with five leaf samples per time point for each genotype. **(C)** Level of ROS. Heat treatment of rice seedlings was performed as in (a). Means and SE were calculated from three experiments with five leaf samples per time point for each genotype. **(D)** Level of MDA. Heat treatment of rice seedlings was performed as in (a). Means and SE were calculated from three experiments with five leaf samples per time point for each genotype. The statistical differences in the *Prx24* transcript level, POD activity, ROS or MDA level between WT and *hst1* mutant plants were tested using a Student *t* test (**P ≤ 0.01).

It has been previously shown that DST binds to the TGCTANNATTG elements in its target gene promoters by the C2H2 zinc finger motif and activates their transcription (Huang et al., 2009; Li et al., 2013b; Cui et al., 2015). Expression profile analysis using the Affymetrix Rice Genome Genechip has identified DST-dependent genes, some of which are related to reactive oxygen species (ROS) homeostasis (Huang et al., 2009). Among the genes directly targeted by DST is *Peroxidase 24 Precursor* (*Prx24*), which encodes a peroxidase that directly scavenges H_2_O_2_ (Huang et al., 2009). To determine whether the expression of *Prx24* was altered in the *hst1* mutant, we compared WT and the *hst1* mutant for the transcript levels of *Prx24* in response to heat stress using qRT-PCR. As shown in **Figure 7A**, the transcript levels of *Prx24* in WT were substantially higher than those in *hst1* even prior to heat treatment. Thus, the loss-of-function mutation in *DST* gene in the *hst1* mutant caused reduction in basal expression levels of *Prx24*. Upon heat treatment at 42°C, we observed rapid reduction in *Prx24* transcripts in both WT and *hst1* mutant during the first 0.5 and 1 hours at the high temperature (**Figure 7A**). Following the rapid reduction during the first hour, the transcript levels of *Prx24* became relatively stable or even increased slightly during the remaining hours of the experiments (**Figure 7A**). Throughout the 12 hours at the high temperature, however, the *Prx24* transcript levels in the *hst1* mutant were generally lower than those in WT, particularly during the first 0.5 to 1 hour under heat stress (**Figure 7A**). We also found that the levels of peroxidase activity was substantially lower in the *hst1* mutant than in WT during the first 24 hours under heat stress (**Figure 7B**).

To determine whether reduced expression of ROS-scavenging *Prx24* was associated with altered ROS levels in the *hst1* mutant, we compared WT and *hst1* for the H_2_O_2_ levels. As shown in **Figure 7C**, the *hst1* mutant had a significantly higher level of H_2_O_2_ than WT even prior to heat treatment. During the first two hours of treat stress, the H_2_O_2_ levels in the *hst1* mutant remained higher than those in WT (**Figure 7C**). By the 12^th^ hour at the high temperature, the H_2_O_2_ levels became similar in WT and *hst1* mutants (**Figure 7C**). Thus, the loss-of-function *hst1* mutation elevated H_2_O_2_ levels prior to and during the early hours of heat treatment. We also compared WT and the *hst1* mutant for the levels of malondialdehyde (MDA), which results from lipid peroxidation of polyunsaturated fatty acids at elevated levels of ROS. Again, we observed that the basal levels of MDA in the *hst1* mutant prior to heat treatment were elevated when compared to those in WT (**Figure 7D**). During the first 6 hours at the high temperature, the MDA level remained significantly different between WT and *hst1* mutant plants (**Figure 7C**). The difference in the MDA levels between WT and the *hst1* mutant during the remaining hours of the experiments was also observed, albeit to a lesser extent (**Figure 7D**).

### Rapid induction of *HSP* gene expression in the *hst1* mutants

**Figure 8:**
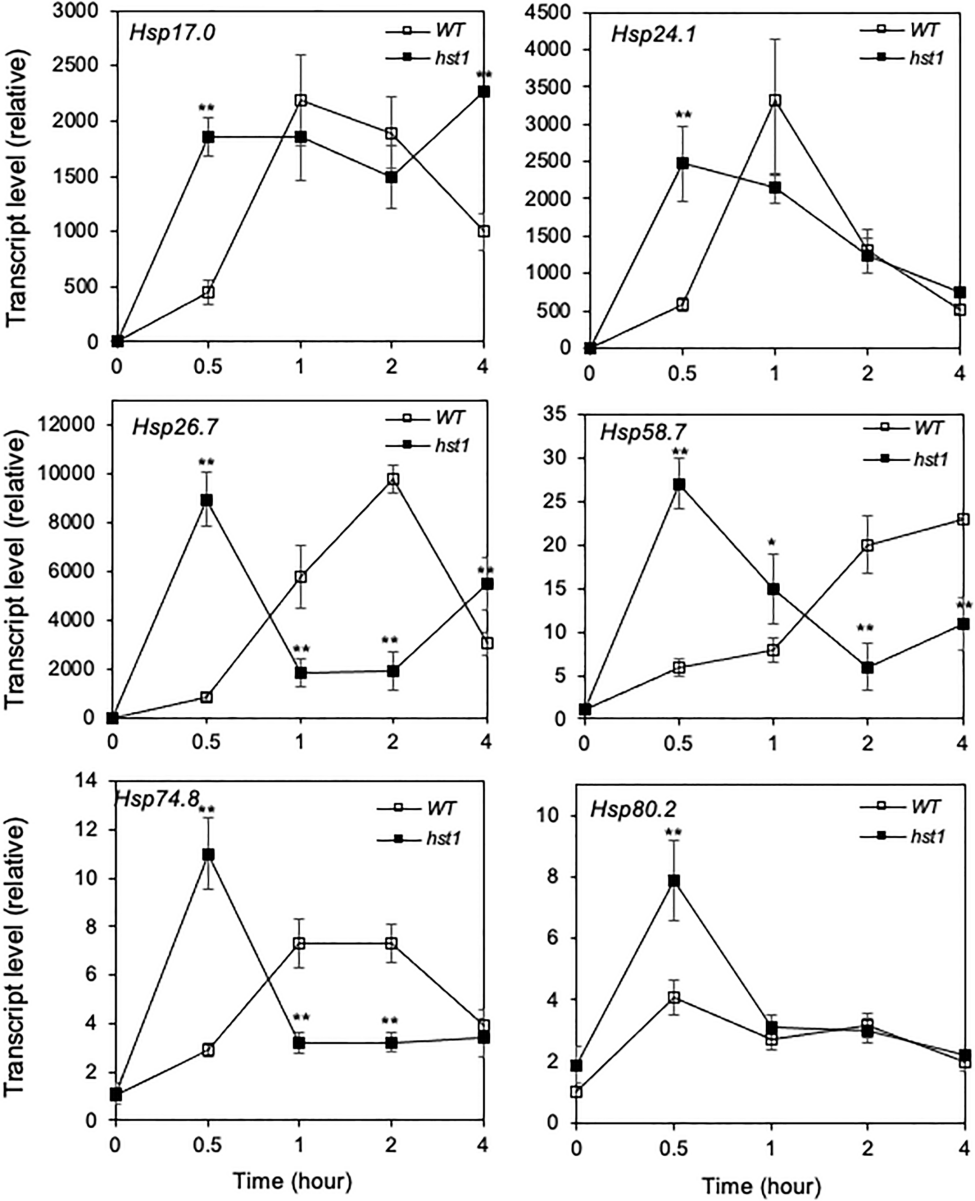
Heat-induced expression of *HSP* genes. WT and *hst1* mutant plants (45-day-old) were placed in a 42°C growth chamber and total RNA was isolated from leaf samples collected at indicated times. Transcript levels were determined using qRT-PCR. Error bars indicate SE (n = 3). The statistical differences in the transcript level between WT and *hst1* mutant were tested using a Student *t* test (*P ≤ 0.05; **P ≤ 0.01).

**Figure 9:**
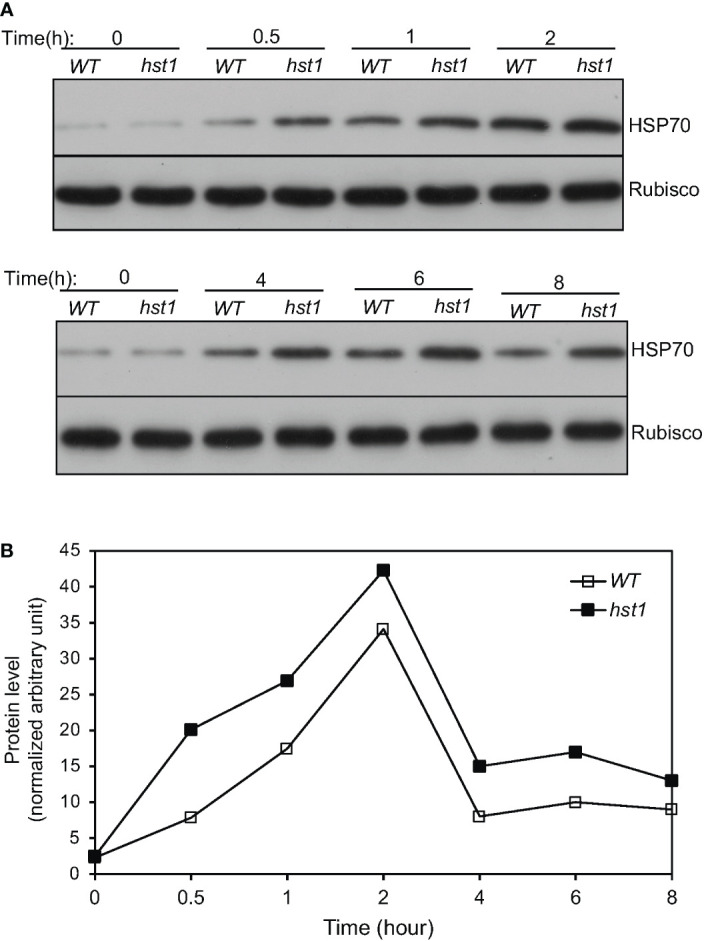
Heat-induced HSP70 accumulation in rice seedlings. **(A)** Ten-day old WT and *hst1* mutant seedlings were placed in a 42°C growth chamber and total proteins were isolated from leaf samples collected at indicated times. The HSP70 protein levels were analyzed by western blotting using anti-HSP70 polyclonal antibodies. Rubisco large subunit proteins, detected with an anti-Rubisco antibody, were used as a loading control. **(B)** HSP70 protein levels at the indicated times of heat treatment were determined from the band intensities of scanned western blots using Image J software. The quantification reflected the relative amounts as a ratio of each HSP protein band to the lane’s Rubisco loading control. The HSP70 protein band intensities from different blots were normalized. The experiments were repeated twice with similar results.

Heat shock responses are found universally in cells that are characterized by rapid transcriptional activation of genes encoding HSPs, many of which act as molecular chaperones in protein quality control (Craig et al., 1993; Jakob et al., 1993). To determine whether enhanced heat tolerance of the *hst1* mutant was also associated with altered expression of genes associated with the heat shock response, we compared 45-day-old WT and the *hst1* mutant for the transcript levels of six *HSP* genes, which have been previously shown to be strongly responsive to heat shock treatment (Zou et al., 2009). In WT, all of these six *HSP* genes displayed rapid induction by heat treatment. Transcript levels for four of the six tested *HSP* genes (*Hsp17.0*, *Hsp24.1*, *Hsp26.7* and *Hsp74.8*) peaked after 1 or 2 hours at the high temperature (**Figure 8**). Rice *Hsp58.7* displayed relatively slow induction and continued to increase in its transcript level after 2 hours of heat treatment (**Figure 8**). Rice *Hsp80.2*, on the other hand, had relatively low induction in its transcript level, although its peak levels occurred quite rapidly at the first 0.5 hour after heat treatment (**Figure 8**). Importantly, the *hst1* mutant was more rapid and robust in induction of these *HSP* genes under heat treatment (**Figure 8**). As a result, the differences in the transcript levels for the six *HSP* genes were most pronounced during the first half hour of heat treatment (**Figure 8**). The transcript levels of five of these six *HSP* genes peaked at 0.5 hour after heat treatment in the *hst1* mutant, which were 0.5 to 2 hour earlier than those in WT (**Figure 8**). Even though they peaked at 0.5 hour after heat treatment in both WT and the *hst1* mutant, the transcript levels of *Hsp80.2* were substantially higher in the *hst1* mutant than in WT at the initial hours of heat stress (**Figure 8**). We also analyzed heat-induced expression of the *HSP* genes in ten-day-old seedlings and found that the *hst1* mutant again displayed significantly faster and stronger induction of the *HSP* genes than WT (**Supplemental Figure 2A**). Furthermore, we performed western blotting using an anti-HSP70 antibody and found that the *hst1* mutant seedlings accumulated HSP70 proteins at rates significantly faster and stronger than those of WT plants during response to heat treatment (**Figure 9**). Thus, enhanced heat tolerance of the *hst1* mutant was also associated with rapid and increased induction of *HSP* gene expression and HSP protein accumulation, which would lead to enhanced heat shock responses.

### Role of ROS in rapid heat induction of HSP genes in the *hst1* mutant

**Figure 10:**
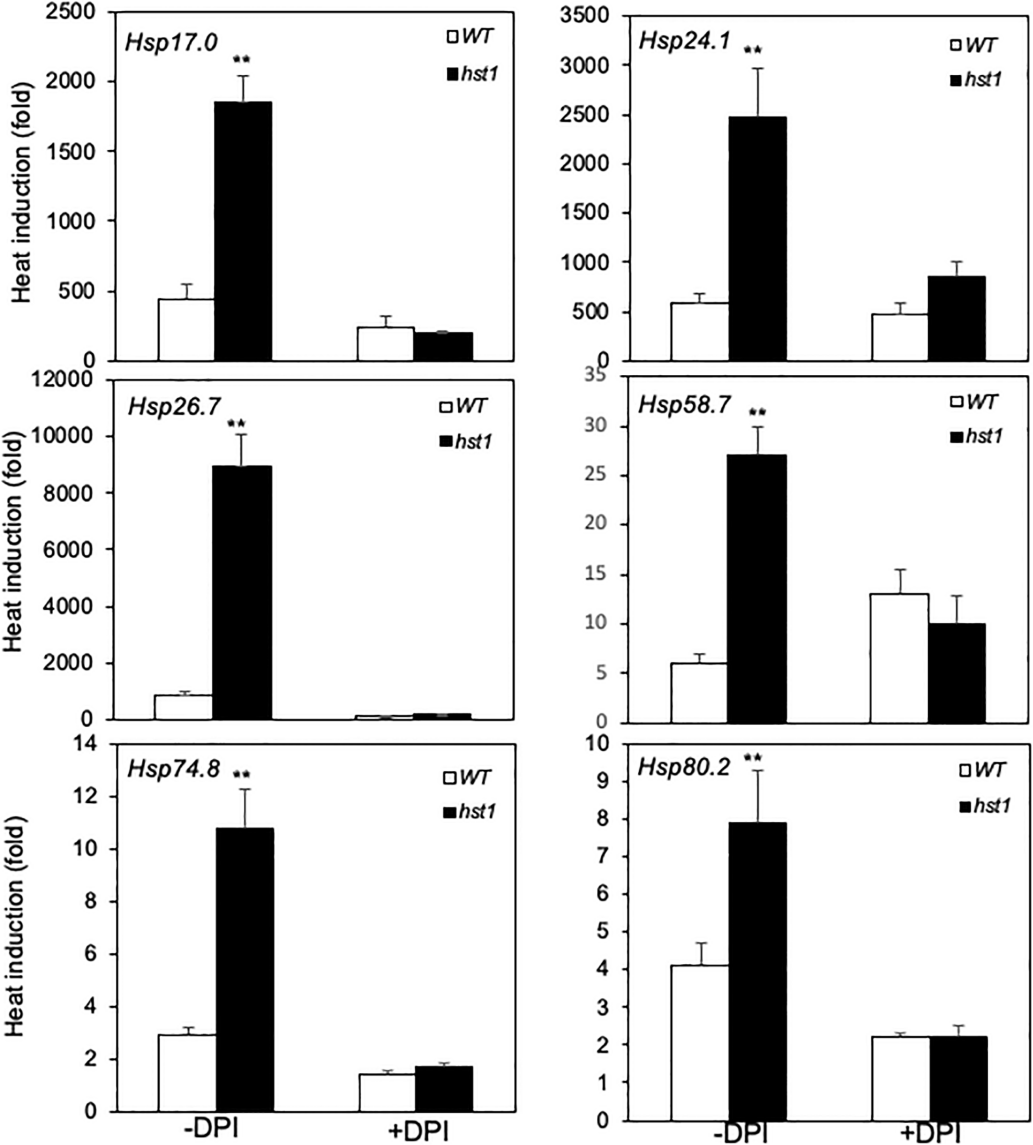
Effect of DPI on early induction of *HSP* gene expression by heat treatment. WT and *hst1* mutant plants (45-day-old) were transferred to the growth medium with (+) or without (-) 10 uM DPI. After 24-hour treatment, the plants were placed in a 42°C growth chamber and total RNA was isolated from leaf samples collected after 0.5 hour of heat treatment. Transcript levels were determined using qRT-PCR. Error bars indicate SE (n = 3). The statistical differences in heat induction of the indicated gene transcript with or without DPI treatment between WT and *hst1* mutant were tested using a Student *t* test (**P ≤ 0.01).

Several studies have shown that H_2_O_2_ is involved in heat shock responses (Miller and Mittler, 2006). Since the *hst1* mutant contained increased levels of H_2_O_2_, we investigated whether the elevated ROS is required for the more rapid and robust induction of rice *HSP* genes. For this purpose, we treated both 45-day-old WT and *hst1* mutant seedlings with diphenyleneiodonium chloride (DPI), an inhibitor of ROS production, and compared WT and *hst1* for the induction of the *HSP* genes during the first half hour of heat treatment, in which the differences in the transcript levels for the six *HSP* genes were most pronounced (**Figure 8**). Indeed, treatment of DPI suppressed the induction of the *HSP* gene transcript levels in the *hst1* mutant to the levels similar to those in WT (**Figure 10**). Similar inhibitory effects of DPI on the stronger induction of *HSP* genes in the *hst1* mutant were also observed at the seedling stage (**Supplemental Figure 2B**). These results suggest that increased ROS levels in the *hst1* mutant are involved in the increased transcription of *HSP* genes in the *hst1* mutant.

### Phenotypes of *hst1* in grain production and heat tolerance at reproductive stages

**Figure 11:**
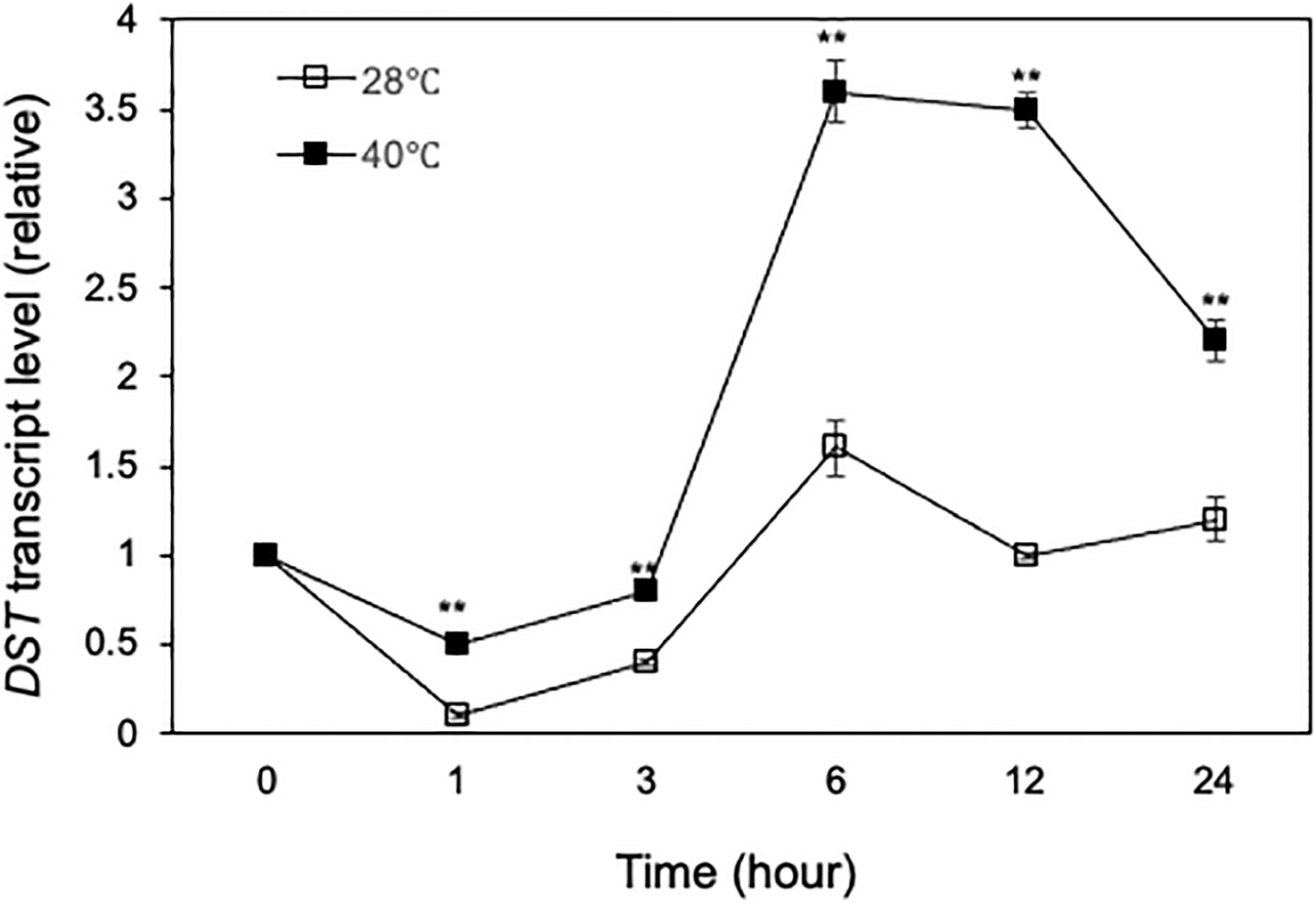
Heat-regulated expression of *DST*. Rice plants on the first day of heading and flowering were placed in a 28°C or 40°C growth chamber and total RNA was isolated from panicle/flower samples collected at indicated times. Transcript levels of *DST* were determined using qRT-PCR. Error bars indicate SE (n = 3). The statistical differences in the transcript levels between 28°C and 40°C were tested using a Student *t* test (**P ≤ 0.01).

Heat stress at the flowering and grain-filling stages seriously affects rice grain quality and yield (Zafar et al., 2020a; Yan et al., 2021; Zafar et al., 2022). To determine the role of DST transcription factor in the rice heat tolerance at reproductive stages, we first analyzed the expression of *DST* in reproductive tissues. Rice plants at the heading and flowering stages were growth at grown at 28°C and 40°C and flowers were collected at various time points for determination of *DST* transcript levels in the reproductive tissues. As shown in **Figure 11**, there was a reduction of DST transcripts during the first hour of treatment at both at 28°C and 40°C. At 28°C, the reduced DST transcripts were largely restored over the next few hours and were largely maintained at the levels similar to those at the beginning of the experiments (**Figure 11**). At 40°C, the *DST* transcripts also recovered from the early reduction and displayed a 3- to 4-fold induction between the 3rd and 6th hours after treatment (**Figure 11**). As a result, the DST transcript levels were 2-4 times higher at 40°C than at 28°C throughout the 12 hours of the experiments (**Figure 11**). Thus, expression of *DST* appears to be more responsive to high temperature in the reproductive tissues than in the seedling leaves (**Figure 4B**).

**Figure 12:**
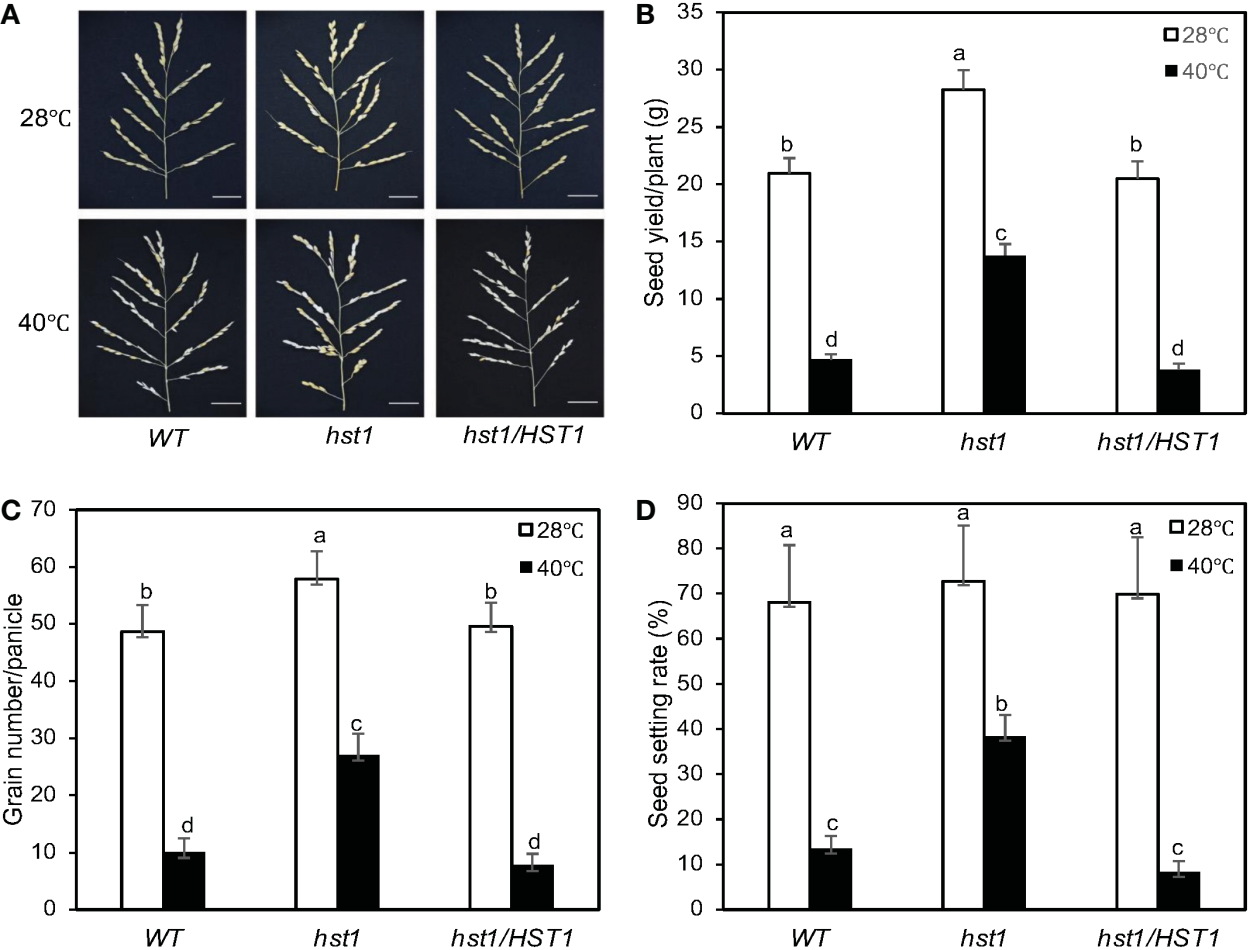
Enhanced reproductive performance of *hst1* under heat stress. Rice plants were moved to a growth chamber one day prior to heading and subjected to a heat treatment regimen (daily 6 hours at 40°C) for 7 days with a 13h light/11h dark photoperiod as described in Materials and Methods. Control plants were grown at 28°C during the 13-hour light period. Plants were grown under normal growth conditions after treatment and their reproductive traits including panicle numbers **(A)**, seed yield per plant **(B)**, grain numbers per panicle **(C)** and seed setting rates **(D)** were evaluated after they reached full maturity. Means and SE were calculated from 40 plants for each genotype. According to Duncan’s multiple range test (P=0.01), means of the reproductive traits do not differ if they are indicated with the same letter. The experiment was repeated three times with similar results.

We also compared grain production between WT and *hst1* mutant grown under the normal and heat stress conditions. Under normal growth conditions, grain production has been previously studied in another rice mutant for DST transcription factor, *reg1*, which contains a single nucleotide insertion that leads to a frameshift after the first 72 amino acids (**Figure 2**). As a result, the NLS and C2H2 zinc finger motif at the N-terminus of DST^reg1^ are not altered but the remaining protein sequence is completely changed (**Figure 2B**) (Li et al., 2013b). The *reg1* mutant is semi-dominant with increased panicle branches and consequently improved grain number (Li et al., 2013b). The *reg1* mutant contained about 14 primary and secondary branches per panicle, compared to about 10 branches in WT plants (Li et al., 2013b). As a result, grain number per main panicle in the *reg1* mutant was 63.8% higher than that of WT plants (Li et al., 2013b). The *hst1* mutant contains a four-base insertion (tggg) between its 194^th^ and 195^th^ nucleotides in DST, which causes a frameshift after the first 64 amino acid residues (**Figure 2**). Therefore, the mutation in the *hst1* mutant not only disrupts the conserved C2H2 zinc finger motif but also alters the remaining protein sequence (**Figure 2**). Intriguingly, the *hst1* mutant had about 10 primary and secondary branches per panicle, similar to those of WT plants (**Figure 12A**). Furthermore, both the seed yield per plant and the grain number per main panicle in the *hst1* mutant were about 20% higher than that of WT, primarily due to significantly higher seed-setting rates in the *hst1* mutant (**Figures 12B, C**). Thus, the beneficial phenotypes of the *hst1* mutant in grain production were not as strong as the *reg1* mutant. Furthermore, unlike the semidominant nature of the *reg1* mutant, the slightly improved grain production in the *hst1* mutant was observed only in the *hst1*/*hst1* homozygous mutant, but not in the *Hst1/hst1* heterozygous plants (**Figures 2A–C**).

To determine the role of DST in rice heat tolerance at reproductive stages, we analyzed the effect of heat treatment on the seed yield of WT and hst1 mutant. As described in Materials and Methods, for heat treatment, rice plants at the heading and flowering stages were subjected to 6-hour daily heat treatment (40°C) during the daytime for 7 days and their seed yields were compared to those of rice plants without the daily heat treatment. As shown in **Figure 12**, under normal conditions, the seed yield per plant of *hst1* was about 20% higher than those of WT and *hst1* complemented with HST1 (*hst1/HST1*). This increase in seed yield in *hst1* was primarily resulted from the increase in the grain number per panicle (**Figure 12**). On the other hand, WT and *hst1* had similar numbers of branches per panicles. Daily heat treatment of 6 hours at 40°C for 7 days reduced seed yield and grain numbers by approximately 80-85% in WT and *hst1/HST1*, but only about 50% in *hst1* (**Figure 12**). Thus, *hst1* mutant was also more tolerant to heat stress at the reproductive stages than WT.

**Figure 13:**
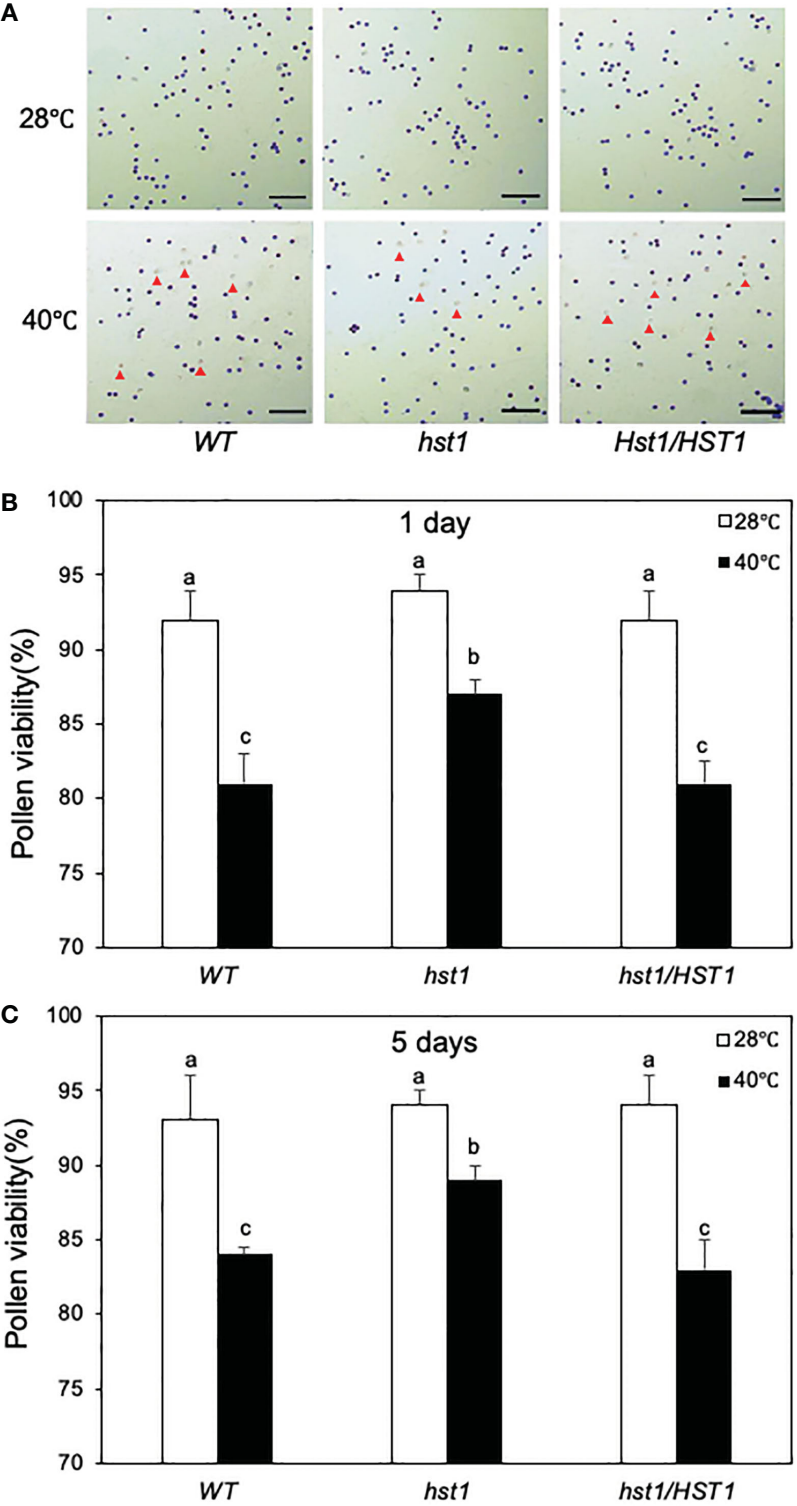
Enhanced pollen viability of *hst1* under heat stress. Rice plants were moved to a growth chamber one day prior to heading and subjected to the same heat treatment regimen as in **Figure 12** and described in Materials and Methods. Pollen viability was assayed with a peroxidase activity staining procedure. Viable pollens stained blue and unviable pollens stained colorless or yellowish as indicated by red markers **(A)**. The percentages of viable pollens after 1 **(B)** and 5 **(C)** days of heat treatment were determined, Means and SE of viable pollen percentages were calculated from approximately 300 pollens from three microscope fields of view. According to Duncan’s multiple range test (P=0.05), viable pollen percentage means do not differ if they are indicated with the same letter. The experiment was repeated three times with similar results.

The negative effects on heat stress on the grain numbers and seed yield in WT and hst1 mutants were strongly correlated with the seed setting rates (**Figure 12D**). Under normal growth conditions, WT, *hst1* and *hst1/HST*1 had very similar seed setting rates of approximately 70% (**Figure 12D**). When grown under daily heat stress, the seed setting rates were reduced by 80-90% in WT and *hst1/HST1*, but only about 45% in *hst1* (**Figure 12D**). We also observed that heat stress has a stronger effect on the pollen viability of WT and *hst1/HST1* than on that of *hst1* (**Figure 13**). Heat treatment for 1 day resulted in reduction of pollen viability by 12% in WT and *hst1/HST1* but only by 7% in *hst1* (**Figure 13**). Interestingly, increasing daily heat treatment from 1 day to 5 days, led to a significant recovery in pollen viability and as a result, the pollen viability of WT and *hst1* was reduced only by 10 and 5%, respectively, when compared to those under normal growth conditions (**Figure 13**). These results collectively indicate that heat stress negatively impact reproductive processes, ultimately leading to reduction in grain formation and seed yield.

### Mutant phenotypes of *hst1* in salt tolerance

As described earlier, two rice mutants, *dst* and *reg1*, for the rice *DST* gene have been previously reported with altered phenotypes in drought and salt tolerance and in grain production, respectively (Huang et al., 2009; Li et al., 2013b; Cui et al., 2015). The *dst* mutant is recessive with increased drought and salt tolerance due to a missense mutation that causes the substitution of an asparagine (N) at residue 69 for a threonine (T) in the conserved C2H2 zinc finger motif (**Figure 2B**). Despite the substitution of a conserved amino acid in the C2H2 zinc finger motif, the sequence-specific DNA-binding activity of the DST^dst^ mutant protein was not altered and the enhanced drought and salt tolerance of the *dst* has been attributed to abolished or reduced transcription-acting activity of the mutant protein (Huang et al., 2009). Therefore, we also examined whether the *hst1* mutant was altered in tolerance to salt. When treated with 0.6% NaCl for 12 days, more than 50% of *hst1* mutant plants but only about 20% WT plants survived (**Supplemental Figure 3**). The enhanced salt tolerance was observed only in the *hst1/hst1* homozygous lines, but not in the *hst1* mutant complemented with the WT DST genomic fragment (*hst1/HST1*). Thus, the *dst* and *hst1* mutants share the same phenotype of enhanced salt tolerance and as with the *dst* mutant, this salt-tolerant phenotype of the *hst1* mutant is recessive. Like the *dst* mutant, the leaf width of the *hst1* mutant was also substantially increased (**Supplemental Figure 4**).

## Discussion

Analysis of two previously reported mutant alleles for DST have shown that the rice C2H2 transcription factor is an important regulator of plant stress tolerance and grain production (Huang et al., 2009; Li et al., 2013b). The *dst* recessive mutant displayed increased drought and salt tolerance due to a missense mutation that results in an amino acid substitution in the conserved C2H2 zinc finger motif (Huang et al., 2009). The semi-dominant *reg1* mutant has increased panicle branches and grain number due to a single nucleotide insertion that causes a frameshift immediately downstream of the C2H2 zinc finger (**Figure 2B**) (Li et al., 2013b). Both DST^dst^ and DST^reg1^ mutant proteins are still capable of sequence-specific DNA-binding (Huang et al., 2009; Li et al., 2013b). Our new *hst1* mutant allele, on the other hand, contains a 4-nucleotide insertion in *DST* that causes a frameshift at the first conserved histidine residue of the C2H2 motif and, as a result, disrupts the zinc finger (**Figure 2**) and abolishes the DNA-binding activity (**Figure 3**). Therefore, unlike the previously reported *dst* and *reg1* mutant alleles, *hst1* is a knockout mutant for the DST transcription factor. Comprehensive analysis of the *hst1* mutant revealed a critical and novel role of the DST transcription factor in rice heat tolerance through both common and heat-specific mechanisms of stress responses. Characterization of the *hst1* mutant also provided important new insights into the regulation of rice grain production that were not obvious from the previously reported *reg1* mutant.

The rice *hst1* mutant seedlings displayed increased heat tolerance based on its increased survival after heat treatment when compared to WT (**Figure 1**). Enhanced heat tolerance of the *hst1* mutant was associated with reduced water loss and wilting during the heat treatment at 42°C (**Figure 5**). Reduced water loss of the *hst1* mutant was further associated with reduced stomatal density and stomatal conductance of the mutant when compared with those of WT (**Figure 5**). These results provided strong evidence that the loss-of-function mutation of the *DST* gene in the *hst1* mutant enhanced heat tolerance in part by reducing heat-induced water loss in leaves. In support of this interpretation, the increased survival rates of the *hst1* mutant after heat treatment over those of WT was strongly influenced by air humidity (**Figure 6**). At 100% air humidity, in which transpirational water loss is eliminated, the survival rates of WT after heat treatment were much closer to those of the *hst1* mutant than at 65% humidity (**Figure 6**). These results point strongly to reduced water stress in the shoots under high temperatures as a critical mechanism for the increased heat tolerance of the *hst1* mutant.

Plant water stress is a critical factor in plant drought tolerance and, therefore, it can be argued that the heat tolerance of the *hst1* is merely a reflection of its drought tolerance. However, drought is a condition of water shortage, which was not present during the heat treatment of WT and the *hst1* mutant plants as they were grown in a large volume of growth medium (4.5 liters) that was replenished daily. Therefore, the reduced stomatal number and reduced stomatal conductance of the *hst1* mutant were likely to be primarily responsible for reduced transpirational water loss, thereby delaying wilting and improving survival under heat stress. Thus, even in the absence of drought, there could still be severe water stress in rice shoots caused by transpirational water loss under heat stress, which can cause severe damage or even death to plants. Accordingly, improving plant ability to reduce water loss under heat stress may be an effective means to improve plant heat tolerance.

The enhanced heat tolerance of the *hst1* mutant broadens the role of the DST C2H2 transcription factor in plant stress responses. Extreme temperature, drought and salinity are among the most important abiotic stresses that negatively impact plant growth, leading to loss of crop yield worldwide. Cross-tolerance to abiotic stresses is well known in plants, whereas exposure to one type of stress often leads to increased tolerance to a range of other abiotic stresses (Rizhsky et al., 2002; Perez and Brown, 2014). Despite the importance and potential in agriculture, the precise molecular mechanisms by which cross-tolerance develops are not fully understood. The demonstrated tolerance of the *dst/hst1* mutants to a broad range of abiotic stresses and shared mechanisms in coping with water stress under different abiotic stresses makes the DST transcription factor highly valuable for studying the molecular and physiological basis of plant cross-tolerance and associated signaling and transcriptional reprograming.

There was increased accumulation of H_2_O_2_ in the *hst1* mutant, associated with reduced expression of genes involved in ROS homeostasis including *Prx2*4 that encodes an important ROS-scavenging enzyme (**Figure 7**) (Huang et al., 2009; Cui et al., 2015). As H_2_O_2_ accumulation is known to regulate stomatal closure (Sierla et al., 2016), it appears that increased H_2_O_2_ as a result of reduced expression of ROS-scavenging genes acts as an early signaling molecule mediating heat-responsive stomatal closure that reduces water loss under high temperature. Interestingly, the rice RING finger ubiquitin E3 ligase OsHTAS, which plays a positive role in heat tolerance at the seedling stage, targets an isoform of rice ascorbate peroxidase, modulate H_2_O_2_ in shoots, alters the stomatal aperture and promotes ABA biosynthesis (Liu et al., 2016). A similar mode of action through modulation of H_2_O_2_ and ABA-mediated stomatal closure has also been proposed for the role of DST in regulation of plant responses to drought and salt (Huang et al., 2009; Cui et al., 2015). The SNAC1-targeted gene *OsSRO1c* modulates stomatal closure and oxidative stress tolerance by regulating H_2_O_2_ in rice (You et al., 2013; You et al., 2014). Therefore, the critical role of ROS in stress-induced and ABA-mediated stomatal closure appears to be a common theme in tolerance to a broad range of abiotic stresses in rice and possibly in other plants as well.

Even at 100% humidity, there was still a significant difference in the relative survival rates between WT and *hst1* mutant plants after heat treatment (**Figure 6**). Furthermore, the *hst1* mutant was more heat-tolerant than WT not only at the seedling stages but also at the reproductive states characterized by increased grain number and seed yield when grown under heat stress conditions. Unlike at seedling stages, even WT plants displayed no wilting symptom at the reproductive stages under the heat stress condition used. Furthermore, the improved grain number and seed yield of the *hst1* mutant relative to those of WT were closely correlated with increased seed setting and pollen viability under high temperature (**Figures 12** and **13**), which cannot be directly attributed to altered stomatal number and behavior. These observations suggests that while leaf water stress plays a major role in increased heat tolerance of the *hst1* mutant, there are other factors that also contribute to its improved fitness and survival of *hst1* under heat stress. Indeed, heat induction of these *HSP* genes was more rapid and robust in the *hst1* mutant than in WT (**Figure 8**; **Supplemental Figure 2A**). Therefore, nuclear heat-responsive gene expression might also play a role in DST-regulated heat tolerance. Treatment with an inhibitor of ROS production reduced heat-induced expression of the *HSP* genes (**Figure 10**; **Supplemental Figure 2B**), indicating that ROS plays a critical role for effective induction of nuclear heat-responsive gene expression. Different classes of HSPs can cooperate to resolubilize protein aggregates after heat stress in plants (Ben-Zvi et al., 2004; Zwirowski et al., 2017). Small HSPs can also stabilize membranes and act as site-specific antioxidants to protect thylakoid membranes against heat stresses in photosynthetic organisms (Heckathorn et al., 2004). Therefore, DST-regulated ROS homeostasis plays an important role in plant heat responses through at least two distinct mechanisms: regulation of stomatal closure to modulate water loss and promotion of nuclear heat-responsive gene expression. Since plant responses to other types of abiotic stresses are also associated with nuclear gene reprogramming, it would be of great interest to determine whether the critical role of DST in plant tolerance to other abiotic stresses also involves ROS-mediated induction of stress-specific genes.

DST has been previously shown to regulate grain production in rice (Li et al., 2013b). This role of DST was revealed from the *reg1* mutant caused by a frameshift immediately downstream of the C2H2 zinc finger of DST (Li et al., 2013b). Our *hst1* mutant was caused by a frameshift in the C2H2 zinc finger of DST (**Figure 2**). As a result, the DST^reg1^ and DST^hst1^ differ only in seven amino acid residues in the C2H2 zinc finger, which is still intact in DST^reg1^ but disrupted in DST^hst1^ (**Figure 2**). The *reg1* mutant contained about 40% more primary and secondary branches per panicle and 63.8% more grains per main panicle than WT plants (Li et al., 2013b). Further analysis has revealed that DST directly regulates rice *Gn1a/OsCKX2* (*Grain number 1a/Cytokinin oxidase 2*) for a cytokinin oxidase in the reproductive meristem (Li et al., 2013b). It has been proposed that with its intact DNA-binding C2H2 zinc finger motif without the C-terminal transcription activation domain, the DST^reg1^ mutant protein acts as a dominant negative regulator that can perturb the direct regulation of *OsCKX2* expression by WT DST proteins in heterozygous *reg1/REG1* plants, leading to the semi-dominant nature of the *reg1* mutant (Li et al., 2013b). According to this model, we would expect that the homozygous *hst1* mutant display the levels of increase in panicle branches and grain number similar to those in the homozygous *reg1* mutant. However, we observed no increase in panicle branches and less than 20% increase in grain number per panicle in the *hst1* mutants (**Figure 11**), compared to about 40% increase in branch number and more than 60% increase in grain number in *reg1* (Li et al., 2013b). The difference in the phenotypes between the two mutants is unlikely to be caused by the different genetic backgrounds since DST^reg1^ can enhance grain production to similar extents in different indica and japonica rice varieties (Li et al., 2013b). We have also sequenced the promoter and coding regions of the *Gn1a/OsCKX2* gene in both WT and the *hst1* mutant and found no mutation (data not shown), thereby excluding the possibility that the relatively weak phenotype of *hst1* in grain production is due to impaired function or expression of the cytokinin catabolic gene. The difference in the phenotypes in grain production between the *reg1* and *hst1* mutants might be caused by different growth conditions. While this possibility cannot be completely ruled out, it is worthy to note that the difference of the *reg1* and *hst1* mutants in grain production was based on their difference to their respective WT plants, which had similar panicle branch and grain numbers from the two studies, suggesting similar growth conditions (Li et al., 2013b) (**Figure 12**). Another possibility for the substantial difference in grain production between the *reg1* and *hst1* mutants could be due to the presence of an additional negative regulator factor of rice grain production that recognizes same sequence as DST or the sequence that overlaps with the DST-binding site in the promoter of *OsCKX2* to activates its expression in reproductive meristem. The DST^reg1^ dominant negative protein would suppress the expression of *OsCKX2* by preventing binding of the *OsCKX2* promoter by WT DST and the new factor. On the other hand, due to lack of DNA-binding activity, DST^hst1^ is unable to prevent binding of the *OsCKX2* promoter by WT DST and the new factor and, therefore, would not inhibit the activation of *OsCKX2*. Thus, the mechanism for the regulation of *OsCKX2* gene expression in grain production could be more complicated than originally thought. Further research on the additional regulators of *OsCKX2* expression in reproductive meristem will help establish the signaling pathways and network of grain production in rice and other grain crops.

In summary, our study has expanded the analysis of HST1/DST as a novel negative regulator of rice tolerance to multiple abiotic stresses including drought, salt and heat stresses. Our comprehensive analysis has also revealed that the broad roles of HST1/DST in abiotic stress tolerance are mediated by both shared and stress-specific mechanisms. In particular, the water status is a critical factor not only for plant tolerance to drought, but also to heat. HST1/DST also negatively regulates rice grain production. These important properties of HST1/DST make it a potential target for genome editing to develop stress tolerant rice with improved yield (Zafar et al., 2020b).

### Accession numbers

The identifiers for the rice genes described in this article are as follows: *HST1* (LOC_Os03g57240), *OsPrx24* (LOC_Os02g06630) and *OsCKX2* (LOC_Os01g10110).

## Data availability statement

The original contributions presented in the study are included in the article/**Supplementary Material**. Further inquiries can be directed to the corresponding authors.

## Author contributions

CZ, YD, ZC, and KY conceived the project. YD, MZ, and KW performed most of the work. AQ, SH, QJ, FW, and CC performed some of the work. YD, CZ and ZC analyzed the data and wrote the paper with input from the other authors. All authors contributed to the article and approved the submitted version.
