# Supplementary material for: Rice DST transcription factor negatively regulates heat tolerance through ROS-mediated stomatal movement and heat-responsive gene expression
Source: Front Plant Sci. 2023 Jan 31;14:1068296. doi: 10.3389/fpls.2023.1068296 (PMC9927019; doi:10.3389/fpls.2023.1068296)
Supplement: Supplementary file 1 [file DataSheet_1.pdf]

Supplemental Table 1. Primers for the molecular markers used in *hst1* mapping.

Marker	Forward primer (5'- 3')	Reverse primer (5' - 3')
M-1	TCGCTTGTGTTTTCTGGGTC	TGGAGAATTTGGAGGCTGC
M-2	AGACCGACCGAACAGACA	ACTCAAGGCCACAGAAGC
M-3	TGGAGGAAGTTGTTGAGGGT	TTTGTTCGGGAATAATGATG
M-4	CAGATGATCAAGTCCCAACC	GCGAGGGGGATTTTATGGTT
M-5	CTGTACTACTCTTCTCTCC	CCCTACGAGATCTTATTCGGT
M-6	GGGTGGTGTGAGCTTTTCTC	TTCCACTTCGACAACCCTTC
M-7	CATAAACCGCTCGGCATTG	CTGACTCACAAGACAACAGGG
M-8	ATTTTTGTAGCGGGCATG	TAAAGAGGAAACTGAAGC
M-9	TAGAGTAGAGTGGGTTTG	TGGTTGAGATGATTGTTA
M-10	ATCCCATCCGCATCCTCC	CACCCGTCACCCGTCAAC
M-11	AACATAGCATAGCAAGCAGA	TTTTAACAAGAGGTAGAACC
M-12	AAGAACCAGAGCAAGATCG G	CTTCCAATTCGATCGCTAGC
M-13	GCCTTTTGTTTTTGCCCA	CCGTCCGTCTTATTTGAA
M-14	CGCTAAGGTAAGGGAGGT	GCGATAGGTTTAAGAAGG
M-15	TGTGAGAGTTCAGAGAGG	AAGCAGAGCTAGGGTTTA
M-16	ATAAGCTACTGCCACCAAGG	ACTGCAATGGATGAAAACCC
M-17	CATCAGGGAAACAGAACAGACCA	GAAGCAGAGGTAATGCCCTAAAA
M-18	TAGGAGAAAAGGAATAAA	ATACAGACCCACCAAGAT
M-19	TTTCATTTTGTTATCGTC	TTGTAAGCAACCTACTCC
M-20	CTTTGTTTCTTCCTCACT	GGCCTCTAGATCAATTGT
M-21	AAGAACCAGAGCAAGATCGG	CTTCCAATTCGATCGCTAGC
M-22	AGGAGAAGCGAGCGAGAA	CCCATTAAGCCTACCAAC

Supplemental Table 2. Primers for RT-qPCR.

Gene	Forward primer (5'- 3')	Reverse primer (5' - 3')
HST1	CGGCTGTTCCCGTGCTTGTTC	CACCCGATGCTCCGCTCCTTC
Prx24	TTTGTGTGCAAGTTCAAGTG	TTGTTGCTGATGTGTCTTGAT
HSP17.0	GGTCACATCGCCAATTCAC	GGTGCCACTTGTCGTTCTT
HSP24.1	TTCCAGGTCAACGTCGAGT	GCACGGTTCTTCCGCTTCA
HSP26.7	AGGTGAGGATGCGGTTCG	CGCTCACAGGCTCACATCC
HSP58.7	TCAACCTCCCCTTTATTACTGC	AACCTTTGGCACCCTCGT
HSP74.8	CGAGCAGTTCGAGTACCAGG	TCAGCCATAGCTTCCCATAC
HSP80.2	CGACGACGAGCAGTATGT	CCAGATGTTCCTCCCAGT
ACTIN	TCCATCTTGGCATCTCTCAG	GTACCCGCATCAGGCATCTG
