# Supplementary material for: Rice DST transcription factor negatively regulates heat tolerance through ROS-mediated stomatal movement and heat-responsive gene expression
Source: Front Plant Sci. 2023 Jan 31;14:1068296. doi: 10.3389/fpls.2023.1068296 (PMC9927019; doi:10.3389/fpls.2023.1068296)
Supplement: Supplementary file 2 [file DataSheet_2.pdf]

Supplemental Figure 1

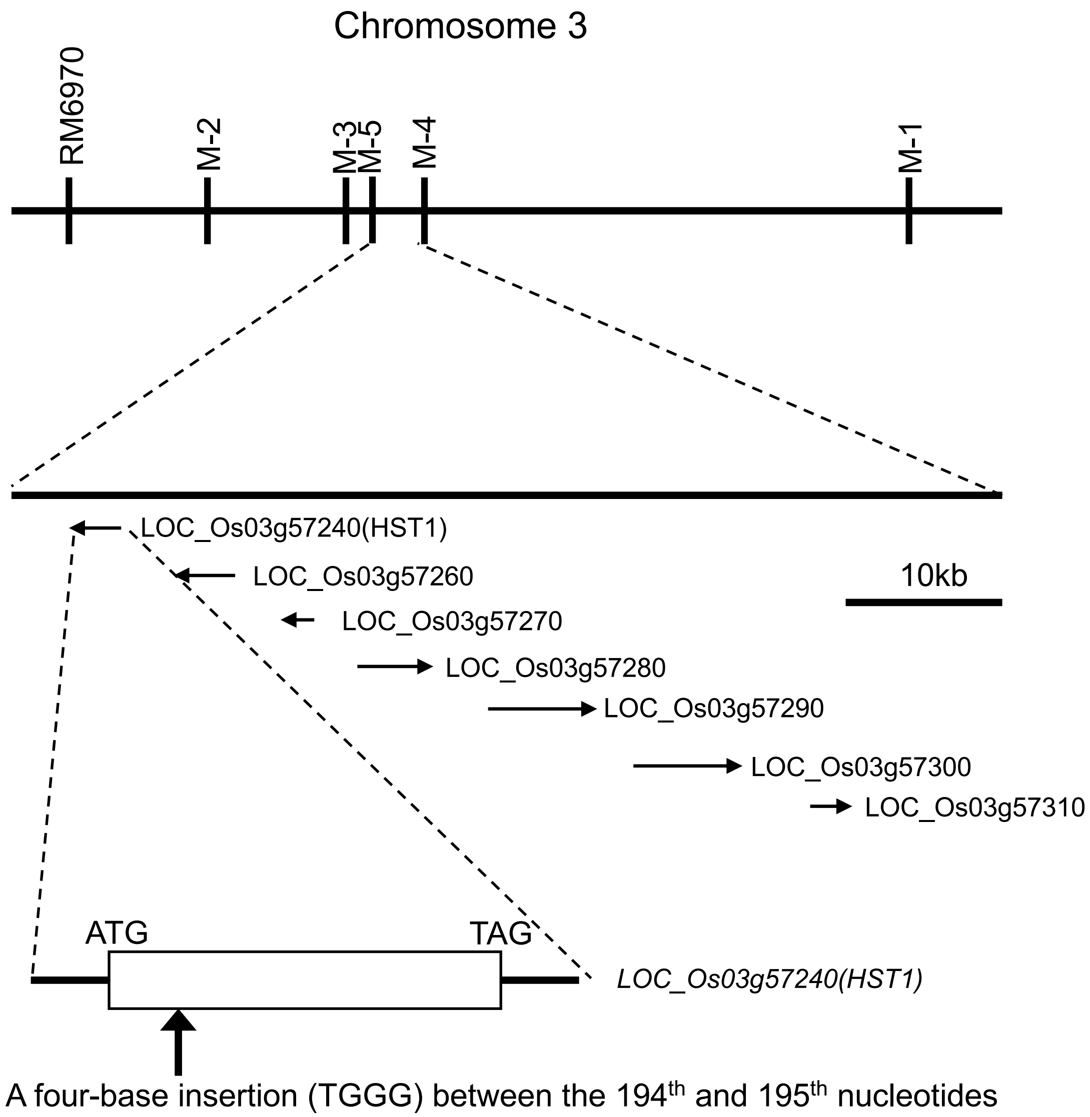

Supplemental Figure 1. Map-based cloning of *HST1*. HST1 was mapped to chromosome 3 and narrowed into a region that contains seven annotated genes. DNA sequencing revealed a four base (TGGG) insertion between the 194th and 195th nucleotides of *LOC_Os03g57240* in the *hst1* mutant.

Supplemental Figure 2

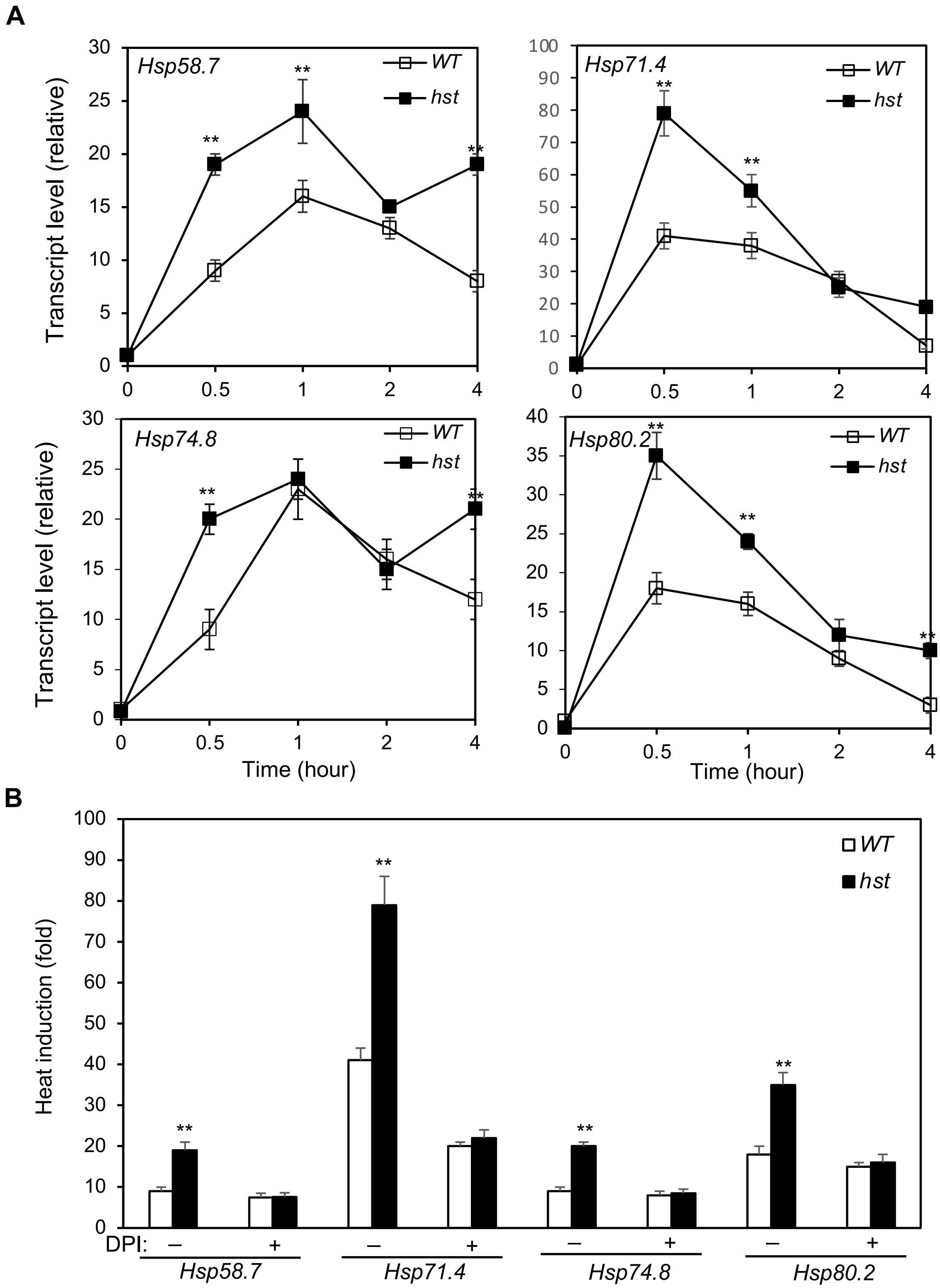

Supplemental Figure 2. Heat-induced expression of *HSP* genes in rice seedlings.

(A) Ten-day old WT and *hst1* mutant seedlings were placed in a 42°C growth chamber and total RNA was isolated from leaf samples collected at indicated times. Transcript levels were determined using qRT-PCR. Error bars indicate SE (n = 3). The statistical differences in the transcript levels between WT and the *hst1* mutant were tested using a Student *t* test (**P ≤ 0.01).

(B) Effect of DPI on early induction of *HSP* gene expression by heat treatment. Ten-day old WT and *hst1* mutant plants were transferred to the growth medium with (+) or without (-) 10 uM DPI. After 24-hour treatment, the plants were placed in a 42°C growth chamber and total RNA was isolated from leaf samples collected after 0.5 hour of heat treatment. Transcript levels were determined using qRT-PCR. Error bars indicate SE (n = 3). The statistical differences in heat induction of the indicated gene transcript with or without DPI treatment between WT and *hst1* mutant were tested using a Student *t* test (**P ≤ 0.01).

Supplemental Figure 3

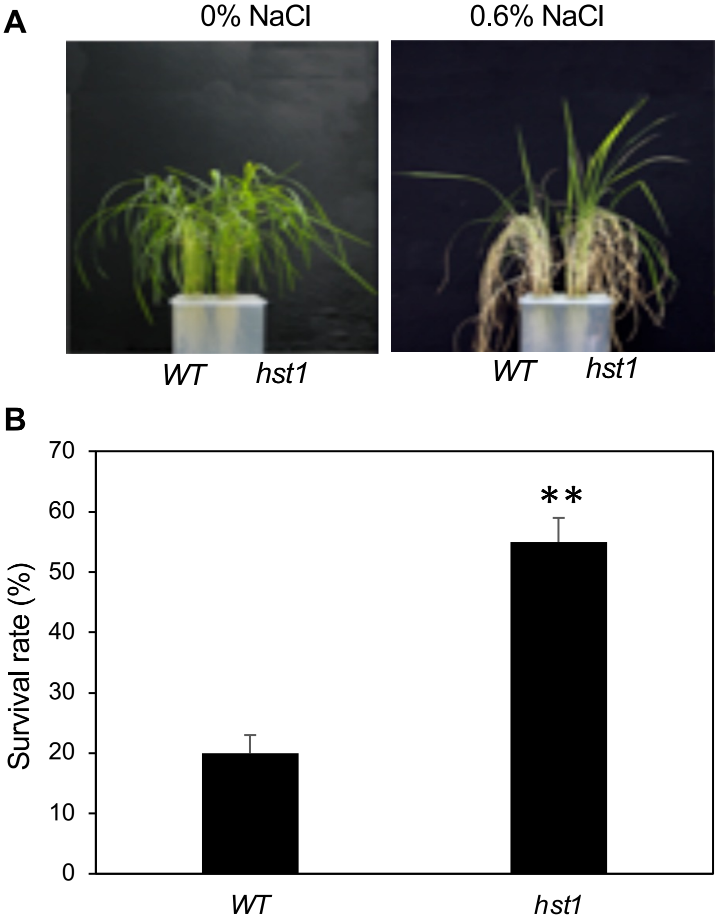

Supplemental Figure 3. Enhanced tolerance of the *hst1* mutant to salt stress.

(A) Picture of WT and *hst1* mutant plants grown in medium containing 0 or 0.6% concentrations of NaCl.

(B) Survival rates of WT and *hst1* mutant plants grown at 0.6% NaCl concentration. The statistical differences in the survival rate between WT and the *hst1* mutant were tested using a Student *t* test (**$P \leq 0.01$).

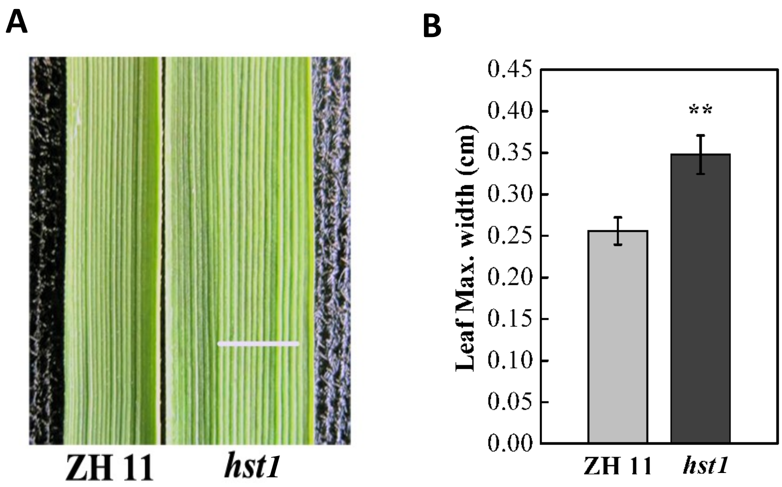

Supplemental Figure 3. Increased leaf width of rice *hst1* mutant.

(A) Leaves 18-day old WT (ZH11) and *hst1* mutant. Bar=0.2 cm.

(B) Maximum leaf widths of WT (ZH11) and *hst1* mutant. Means and SE were calculated from 20 leaves of 18-day old seedlings. The statistical difference in the maximum leaf width between WT and *hst1* mutant was tested using a Student *t* test (**$P \leq 0.01$).
